# Epsilon toxin–producing *Clostridium perfringens* colonize the multiple sclerosis gut microbiome overcoming CNS immune privilege

**DOI:** 10.1172/JCI163239

**Published:** 2023-05-01

**Authors:** Yinghua Ma, David Sannino, Jennifer R. Linden, Sylvia Haigh, Baohua Zhao, John B. Grigg, Paul Zumbo, Friederike Dündar, Daniel Butler, Caterina P. Profaci, Kiel Telesford, Paige N. Winokur, Kareem R. Rumah, Susan A. Gauthier, Vincent A. Fischetti, Bruce A. McClane, Francisco A. Uzal, Lily Zexter, Michael Mazzucco, Richard Rudick, David Danko, Evan Balmuth, Nancy Nealon, Jai Perumal, Ulrike Kaunzner, Ilana L. Brito, Zhengming Chen, Jenny Z. Xiang, Doron Betel, Richard Daneman, Gregory F. Sonnenberg, Christopher E. Mason, Timothy Vartanian

**Affiliations:** 1 Feil Family Brain and Mind Research Institute,; 2 Jill Roberts Institute for Research in Inflammatory Bowel Disease,; 3 Joan and Sanford I. Weill Department of Medicine, and; 4 Department of Microbiology and Immunology, Weill Cornell Medical College, Cornell University, New York, New York, USA.; 5 Immunology and Microbial Pathogenesis Program and; 6 Applied Bioinformatics Core, Division of Hematology/Oncology, Department of Medicine, Institute for Computational Biomedicine, Weill Cornell Medicine, New York, New York, USA.; 7 Physiology and Biophysics, Institute for Computational Biomedicine, Weill Cornell Medical College, Cornell University, New York, New York, USA.; 8 Departments of Pharmacology and Neurosciences, UCSD, San Diego, California, USA.; 9 Harold and Margaret Milliken Hatch Laboratory of Neuro-endocrinology and; 10 Laboratory of Bacterial Pathogenesis and Immunology, Rockefeller University, New York, New York, USA.; 11 Department of Neurology, Weill Cornell Medical College, Cornell University, New York, New York, USA.; 12 Department of Microbiology and Molecular Genetics, University of Pittsburgh School of Medicine, Pittsburgh, Pennsylvania, USA.; 13 California Animal Health and Food Safety Laboratory, School of Veterinary Medicine, UCD, Davis, California, USA.; 14 Astoria Biologica Inc., Norwalk, Connecticut, USA.; 15 Meinig School of Biomedical Engineering, Cornell University, Ithaca, USA.; 16 Division of Biostatistics, Department of Population Health Sciences, and; 17 Genomics Resources Core Facility, Core Laboratories Center, Weill Cornell Medicine, New York, New York, USA.

**Keywords:** Autoimmunity, Microbiology, Demyelinating disorders, Multiple sclerosis, Toxins/drugs/xenobiotics

## Abstract

Multiple sclerosis (MS) is a complex disease of the CNS thought to require an environmental trigger. Gut dysbiosis is common in MS, but specific causative species are unknown. To address this knowledge gap, we used sensitive and quantitative PCR detection to show that people with MS were more likely to harbor and show a greater abundance of epsilon toxin–producing (ETX-producing) strains of *C*. *perfringens* within their gut microbiomes compared with individuals who are healthy controls (HCs). Isolates derived from patients with MS produced functional ETX and had a genetic architecture typical of highly conjugative plasmids. In the active immunization model of experimental autoimmune encephalomyelitis (EAE), where pertussis toxin (PTX) is used to overcome CNS immune privilege, ETX can substitute for PTX. In contrast to PTX-induced EAE, where inflammatory demyelination is largely restricted to the spinal cord, ETX-induced EAE caused demyelination in the corpus callosum, thalamus, cerebellum, brainstem, and spinal cord, more akin to the neuroanatomical lesion distribution seen in MS. CNS endothelial cell transcriptional profiles revealed ETX-induced genes that are known to play a role in overcoming CNS immune privilege. Together, these findings suggest that ETX-producing *C*. *perfringens* strains are biologically plausible pathogens in MS that trigger inflammatory demyelination in the context of circulating myelin autoreactive lymphocytes.

## Graphical abstract

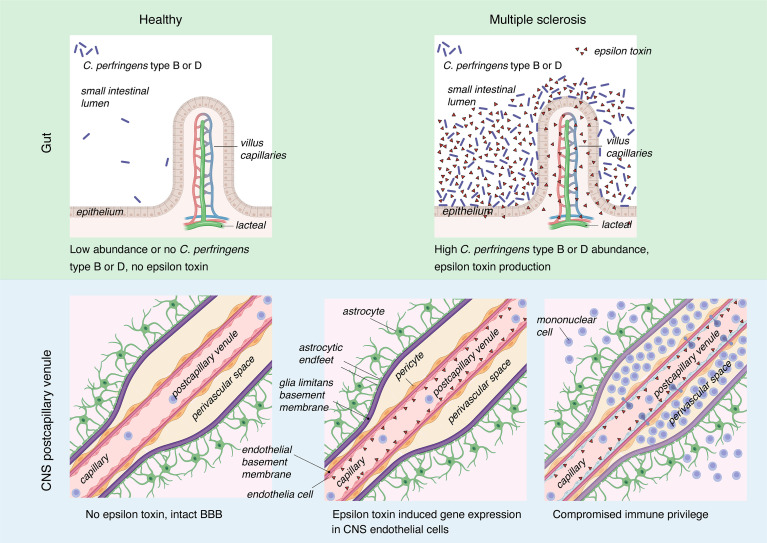

## Introduction

Multiple sclerosis (MS) lesions are centered on postcapillary venules (1, 2), and impairment of blood-brain barrier (BBB) function is considered the earliest event in lesion evolution, paving the way for entry of myelin autoreactive lymphocytes (2–4). MS disease initiation, and thus lesion formation, is believed to require an environmental trigger in a genetically susceptible individual, but biologically plausible environmental agents responsible for lesion induction have been elusive (5, 6). Recent studies have consistently identified dysbiosis in patients with MS, based on taxonomic and functional features of the gut microbiome (7–19). Transfer of gut microbiota from identical twins discordant for MS revealed a significantly higher incidence of autoimmunity in mice receiving microbiota from MS-affected mice compared with their healthy twins (9). Several studies in mice provide compelling evidence that the MS gut microbiome harbors microbes that directly act on adaptive immune cells to enhance or suppress autoimmunity (9, 12, 20–25). Epsilon toxin (ETX) produced by *C*. *perfringens* is a unique candidate environmental trigger for MS because this bloodborne neurotoxin specifically targets CNS endothelial cells, leading to disruption of BBB integrity (26).

### C.

*perfringens* is a gram-positive anaerobe categorized into 7 toxinotypes based on production of 1 or more of 6 major toxins (27). The type B and D strains carry the plasmid-encoded ETX gene (*etx*) (27). In the gastrointestinal tract of mammals, *C*. *perfringens* types B and D exist in the small intestine, where they produce ETX episodically during log-phase growth (28–30). Human exposure to *C*. *perfringens* strains is broad; they are present in our food chain and pets, and are found in multiple ecological niches including farm runoff, sewage, marine sediment, soil, and the gastrointestinal tracts of fish, birds, and mammals (31–43). As a spore-forming anaerobe, once *C*. *perfringens* enters an environment it tends to persist due to the spores’ resistance to heat, chemicals, radiation, and pressure (44–46). Following environmental exposure and oral ingestion, colonization of the small intestine is thought to be dependent on host genetics, microbiome composition, and additional factors such as prior antibiotic use (47).

ETX is a member of the aerolysin-family of pore-forming toxins (48–50). ETX is synthesized as a 32.9 kDa inactive protoxin that is cleaved in the gut by host serine proteases and carboxypeptidases (48, 51), or, occasionally, by bacterially derived lambda protease, into a 27 kDa active toxin (48, 52). The ETX monomer crosses the small intestine without causing injury or enteritis (53). We identified the myelin and lymphocyte protein (MAL) as the receptor for ETX (54), which is required for binding and for all known biologic activity (26, 54–58). MAL is localized to lipid rafts, and ETX binding to MAL brings monomers into proximity, favoring self-assembly into a heptameric prepore complex that then inserts into the plasma membrane of host cells (59–61). In the bloodstream, ETX has access to all vascularized tissues, but its binding is restricted to CNS endothelial cells, since they are enriched for expression of the ETX receptor, MAL (26). In vivo, ETX induces BBB permeability (26, 56, 62–64).

In this report, we present evidence that ETX-producing strains of *C*. *perfringens* can function as environmental triggers for MS. To characterize the possible role of ETX in MS, we (a) identified an association between ETX-producing strains of *C*. *perfringens* and MS in clinical samples; (b) found that people with MS have increased abundance of ETX-producing strains of *C*. *perfringens* in their gut microbiome compared with healthy controls; (c) showed that ETX was sufficient to induce multifocal, inflammatory demyelination in the context of active immunization with MOG_35-55_; (d) demonstrated that ETX-induced inflammatory demyelination in active EAE more closely resembled the lesion distribution observed in MS when compared with the traditional PTX model; and (e) found that, in CNS endothelial cells, ETX induced expression of genes known to overcome CNS immune privilege.

## Results

### The MS gut microbiome harbors ETX-producing C. perfringens strains.

Based on a statistical power calculation (Methods), 62 participants were recruited under the IRB protocol: Harboring the Initial Trigger of Multiple Sclerosis (HITMS), consented, and received instruction on self-collection of fecal samples. Participants who were healthy controls (HCs) and those with MS were matched for age, gender, BMI, location of residence at time of fecal sample donation, and ancestry or ethnicity (Supplemental Table 1 and Supplemental Figure 1; supplemental material available online with this article; https://doi.org/10.1172/JCI163239DS1). Inclusion into the MS arm of the study required a diagnosis of confirmed MS based on the 2010 revised McDonald criteria (65). Participants having a first- or second-degree relative with a diagnosis of MS or a clinically isolated syndrome were excluded from the HC arm. Full inclusion and exclusion criteria are detailed in the Methods and Supplemental Methods.

**Figure 1:**
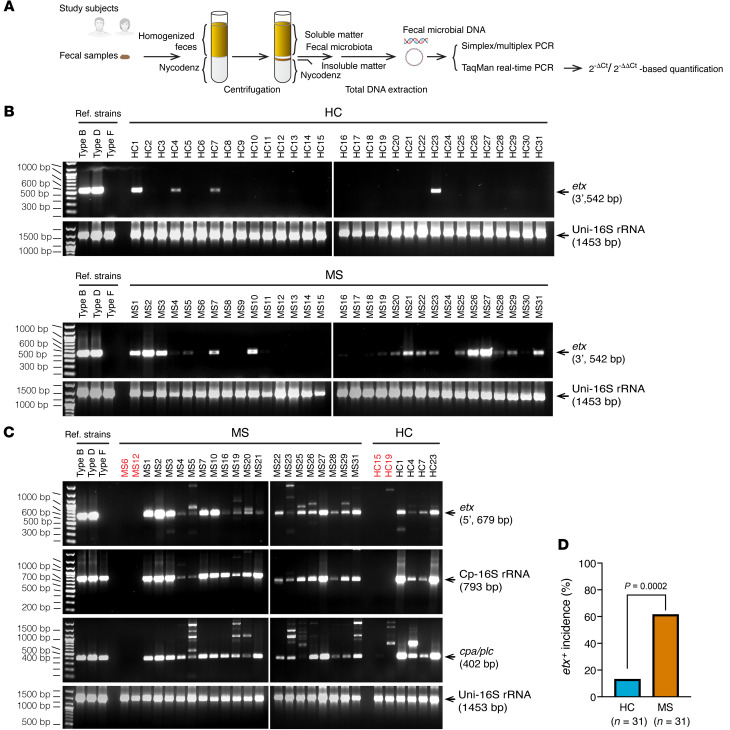
Prevalence of *etx* in the gut microbiome of people with MS and people who were HCs. (**A**) Workflow of the experimental setup. Fecal bacteria were purified by density gradient centrifugation in a Nycodenz solution. (**B**) Initial screen by PCR targeting the 3′-terminal sequence of *etx* (542 bp) detected more frequent presence of *etx* in people with MS compared with people who were HCs. (**C**) Confirmatory assays for *etx*^+^ participants from the initial screen (**B**) using PCR for *C*. *perfringens*-specific genes. PCRs targeting the 5′-terminal sequence of *etx* (679 bp) confirmed the initial detection results. Detection of *C*. *perfringens*-specific 16S rRNA and the chromosomally located alpha toxin (*cpa/plc*) gene present in all *C*. *perfringens* strains corroborates the source of the *etx* gene. Participants labeled in red are included as known *etx*^–^ samples from **B** and included for comparison. Reference strains including *C*. *perfringens* type B (ATCC3626), type D (FD203), and type F (ATCC12915) were included as additional controls. Types B and D are *etx*-harboring strains and type F is negative for *etx*. Types B, D, and F are all positive for *cpa/plc* and *C*. *perfringens*-specific 16S rRNA. (**D**) Statistical analysis of *etx* frequency by Fisher’s exact test. Similar results were achieved in 3 independently repeated PCR experiments using the same people.

We recognized that ETX-producing strains of *C*. *perfringens* would likely be in relative low abundance in human fecal samples based on the results of previous surveys (57, 66) and because the ecological niche of these mucosal-adherent bacteria is in the small intestine (67), a site known to be vastly underrepresented in fecal samples (68, 69). Low abundance in fecal samples likely renders *C*. *perfringens* types B or D undetectable by metagenomics sequencing commonly utilized in MS microbiome studies (9, 12). We therefore utilized PCR, a more sensitive approach for gene detection than shot-gun metagenomics, to examine whether the *etx* gene is present in human gut microbiota. We used a density gradient centrifugation, Nycodenz, in our study because separation of bacteria from nonmicrobial fecal matter removes PCR inhibitors, provides a more accurate representation of bacterial composition, and enhances recovery of the phylum Bacillota (Firmicutes), and, in particular the class Clostridia (70) (Figure 1A). Our initial screen detected an occurrence of *etx* in 61% of MS patients and 13% of controls (Figure 1B; see complete unedited blots in the supplemental material.). To confirm *etx*-positive study participants, we conducted an independent PCR, targeting a different region of the *etx* gene (Figure 1C). The identities of all PCR products were verified by Sanger sequencing. The *cpa/plc* gene, common to all *C*. *perfringens* strains, and the 16S ribosomal RNA gene specific to *C*. *perfringens,* were also detected in those *etx*^+^ participants (Figure 1C), as anticipated, since only *C*. *perfringens* are known to naturally encode the *etx* gene. A Fisher’s exact test of independence showed that *etx* was significantly associated with disease status (MS versus HC), with *P* = 0.0002 (Figure 1D). The odds ratio of *etx-*positivity, comparing people with MS and people categorized as HCs, was 10.7, with a 95% CI of 2.9871–38.2381 and *P* = 0.0003. We further conducted a multivariable logistic regression to determine if disease status (MS diagnosis or healthy control) was associated with etx status (*etx*^–^ or *etx*^+^), independent of sex, with or without stratification by treatment status (treated or not treated). We found that the disease status was independently associated with *etx* status, even when adjusting for sex. In addition, the disease status (MS versus HC), with or without treatment, was independently associated with *etx* status, adjusting for sex (Supplemental Table 2).

The prevalence of fecal *etx* positivity in our analysis was consistent with a previously reported frequency of ETX immunoreactivity in sera from people with clinically definite MS (43%) and age-matched controls (16%) (71). This suggests that there is a high translation rate of the *C*. *perfringens* type B/D virulence factor, *etx*, and that the human gastrointestinal tract is a suitable environment for *C*. *perfringens* type B/D growth.

**Figure 2:**
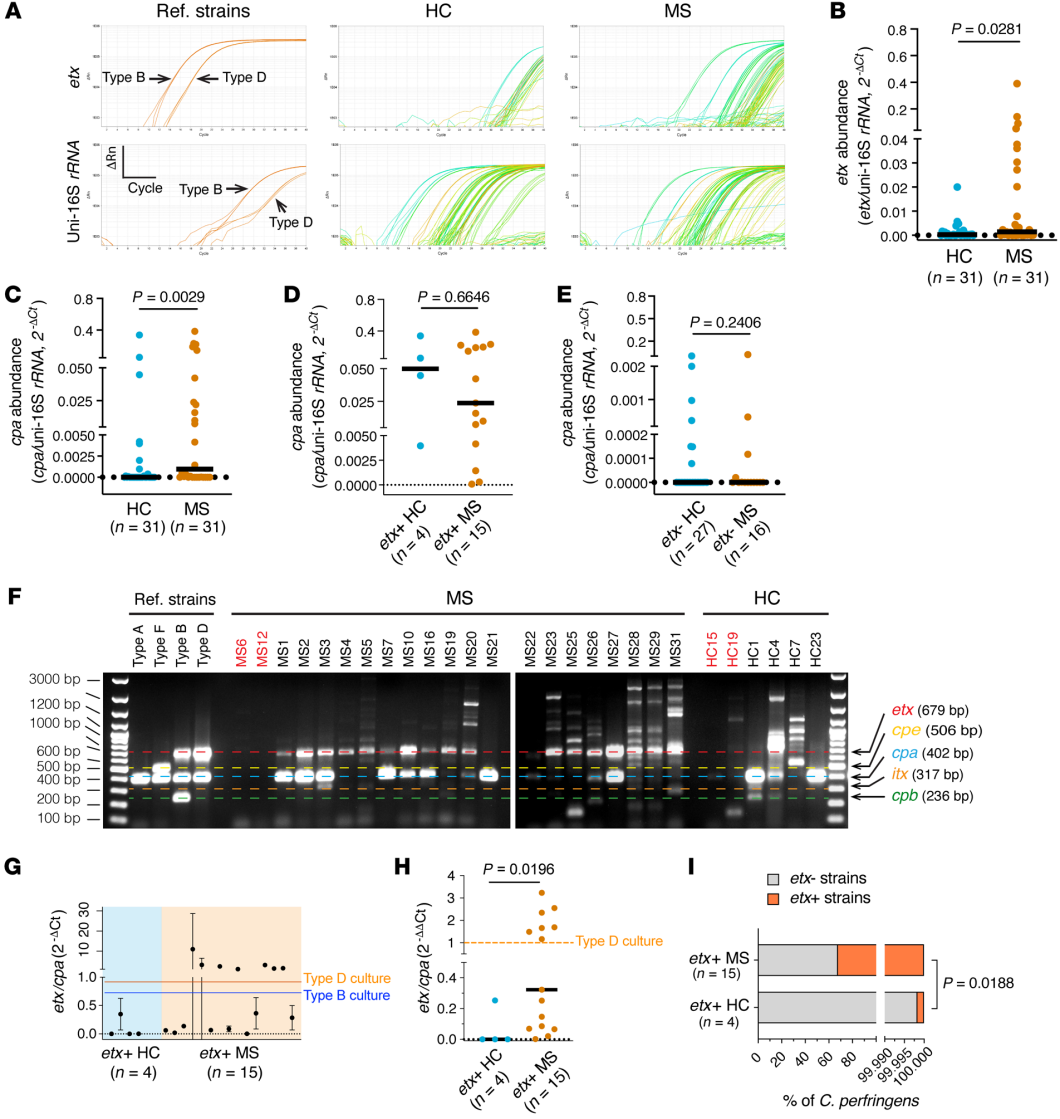
*etx* abundance and *etx*-harboring strains increase in the fecal microbiota of patients with MS. (**A** and **B**) Representative amplification plots (**A**) and statistical analysis of *etx* abundance (**B**) by TaqMan real-time PCR analysis. Of note, the system detected 15 *etx*^+^ patients with MS, fewer than what was detected by standard PCR (19). (**C**–**E**) *cpa* abundance in people who were healthy controls (HCs) and patients with MS. (**F**) Multiplex PCR reveals different composition of strains in the fecal *C*. *perfringens* community from participants who were *etx*^+^. Participants labeled in red are included as known *etx*^–^ samples from (1B) and included for comparison. Of note, *C*. *perfringens* type E, defined by the presence of *cpa* and *itx*, is present in MS3, MS31, and HC1; while MS19, MS26, HC1, and HC4 carry *cpb*, a virulence determinant carried by *C*. *perfringens* types C and B. (**G**–**I**) Analysis of the *etx*/*cpa* ratio by TaqMan real-time PCR using the *C*. *perfringens* type B (ATCC3626) and type D (FD203) strains as a calibrator for the quantification of *etx*^+^/*etx^–^* strain ratios in the fecal microbiota from *etx*^+^ participants. Quantification of the ratio of *etx*/*cpa* by 2^–ΔCt^ (**G**), *etx*/*cpa* against type D culture as calibrator by 2^–ΔΔCt^ (**H**). Dashed horizontal lines in **G** and **H** show 2^–ΔCt^/2^–ΔΔCt^ values from culture of indicated reference strains. Estimate of maximum of percentages of *etx^–^* and *etx*^+^ strains in *etx*^+^ HC and MS fecal microbiome (**I**). The estimates assume that the individuals with a 2^–ΔΔCt^ value above 1 (type D) contain 100% *etx*^+^ strains. *P* value determined by Mann-Whitney test (non-Gaussian distribution). Number of participants (n) is indicated in individual panels; black lines in **B**–**E**, and **H** indicate medians. Data in **G** are mean ± SD, *n* = 3 technical triplicates in a representative real-time PCR experiment). Similar results were achieved in 3 independently repeated PCR/qPCR experiments using the same participants.

We next assessed the relative abundance of *etx* in fecal microbiota harvested from people with MS and HC. To do this, we performed quantitative analysis of *etx* abundance relative to universal *16S* by TaqMan real-time PCR using a modified 2^–ΔCt^ method (72). The 2^–ΔCt^ method allows for normalization of real-time, quantitative PCR data to an internal reference. We assessed the abundance of *etx* relative to universal *16S* rRNA in MS and HC and found that *etx* abundance was significantly increased in fecal microbiota harvested from people with MS compared with people who were HCs (Figure 2, A and B).

### C.

*perfringens* type A strains, defined by presence of the chromosomally encoded α toxin gene, *cpa*, but none of the other major toxin genes, are human intestinal commensals (27, 73). *C*. *perfringens* type B strains carry the *cpa*, *cpb*, and *etx* toxin genes, whereas *C*. *perfringens* type D strains carry the *cpa* and *etx* genes (27). Our PCR analysis identified *cpa* in 74% of patients with MS compared with 45% of people who were HCs (Supplemental Figure 2, A–C). When *cpa* prevalence was analyzed in subgroups of participants, according to the presence or absence of *etx*, *cpa* coexisted with the *etx* gene in participants who were *etx*^+^ (Supplemental Figure 2D). Interestingly, *cpa* prevalence among participants who were *etx*^–^ was essentially indistinguishable between people with MS and people who were HCs (Supplemental Figure 2D). These results suggest non-*etx* strains (*cpa*^+^, *etx^–^*) are unlikely to be involved in MS. TaqMan real-time PCR showed a significant increase in *cpa* abundance for people with MS compared with people who were HCs (Figure 2C). No statistical difference for *cpa* abundance was found among subgroups according to *etx* presence/absence (Figure 2, D and E). Neither prevalence nor abundance of *cpa* from participants who were *etx*^–^ differed between individuals with MS and people who were HCs, ruling out a role for non-*etx* strains in MS pathogenesis. However, an increase in *cpa* prevalence in the MS group (*etx*^–^ and *etx*^+^ combined) compared with the HC group (Figure 2C) suggests that people with MS may have a more suitable gastrointestinal environment for the survival and growth of *C*. *perfringens*.

Quantification by TaqMan real-time PCR simultaneously targeting genes encoding *C*. *perfringens*–specific 16S rRNA and universal 16S rRNA confirmed an increase of *C*. *perfringens* abundance in people with MS compared with people who were healthy controls (Supplemental Figure 3A). Using the 2^–ΔΔCt^ method (72) with pure reference strains as calibrators, participants with MS trended toward higher percentages of *C*. *perfringens* in fecal microbiota when compared with people who were healthy controls, but this did not achieve statistical significance (Supplemental Figure 3, B–D). Of note, our estimates agree with the previously reported range (0.000001%–0.01%) of *C*. *perfringens* abundance in fecal microbiota from healthy humans, confirming the organism’s low abundance (66, 74).

The 2018 revised classification scheme for *C*. *perfringens* defines 7 distinct toxinotypes based on carrying 1 or more of 6 major toxin genes (*plc/cpa, cpb, etx, iap/iab, cpe,* and *netB*) (27). We performed multiplex PCR for the major toxins used to characterize *C*. *perfringens* toxinotypes within our *etx*^+^ HC and MS populations (Figure 2F) (27). The majority of patients with MS who were *etx*^+^ harbored *C*. *perfringens* type D strains (*etx*^+^, *cpa*^+^) with fewer type B (*etx*^+^, *cpa*^+^, *cpb*^+^) strains detected (Figure 2F). *C*. *perfringens* enterotoxin (*cpe*), which is the main agent of *C*. *perfringens*–mediated human food poisoning, was not detected. Notably, unlike the more sensitive singleplex PCR (Figure 1C), *etx* and *cpa* appeared to be absent in some participants by multiplex PCR, likely due to issues of annealing temperature compatibility among individual primer pairs in the multiplex PCR system. The apparent variations of *etx*/*cpa* band intensity ratios among participants, which differ from that of reference strains, suggested that some participants likely harbored a community of combined type A strains (*cpa*^+^) and type B/D strains (*cpa*^+^*, cpb*^+^*, etx*^+^ or *cpa*^+^*, etx*^+^).

We next examined the significance of *etx*-positivity in the individuals who were HCs. Our multiplex PCR result suggested that there was a distinct lack of or weakened *etx* band in the group of 4 etx^+^ HC participants (Figure 2F), despite *etx* being clearly detected in singleplex PCR (Figure 1, B and C). To understand this phenomenon, we developed a sensitive TaqMan real-time PCR to simultaneously target *etx* and *cpa* genes and used the 2^–ΔΔCt^ analysis to quantify the relative abundance of *etx*-producing (*cpa*^+^, *etx*^+^) over non-*etx–*producing strains (*cpa*^+^, *etx*^–^) from *etx*^+^ MS and HC groups. The 2^–ΔCt^ value (Figure 2G) was computed to quantify fold changes of the *etx*/*cpa* ratio when a pure type D (*cpa*^+^, *etx*^+^) culture was used as a calibrator (Figure 2H). A value of 1 was set as the 2^–ΔΔCt^ for the type D calibrator. We reasoned that a 2^–ΔΔCt^ value greater than 1 indicated the dominance of *etx*-encoding strains with increased *etx*-plasmid copy numbers*,* and that a 2^–ΔΔCt^ value of less than 1 indicated a higher percentage of *C*. *perfringens* type A (*cpa*^+^) in the bacterial community. Our results revealed significantly increased 2^–ΔΔCt^ values for the *etx/cpa* ratio in people with MS compared with people who were HCs (Figure 2H).

Since a 2^–ΔΔCt^ value of at least 1 would suggest that 100% of a participant’s *C*. *perfringens* strains were *etx*-harboring (i.e., type B and/or type D), we then obtained a maximum estimate of the average percentage of *etx*-harboring strains in the *C*. *perfringens* community for people with MS or people who were HCs. Our analysis showed that participants with MS who were *etx*^+^ had 32% *etx*-harboring strains and 68% non-*etx* *C*. *perfringens* strains (Figure 2I). In contrast, people who were healthy controls but were *etx*^+^ had 0.002% *etx*-harboring strains and nearly 100% non-*etx* *C*. *perfringens* strains. Analysis from a different perspective by quantifying the abundance of *etx* over *C*. *perfringens-*specific 16S rRNA yielded a higher estimate of the percentage of *etx*-harboring strains; 43% in people with MS and a lower percentage, 0.001%, in people who were healthy controls (Supplemental Figure 4).

Together, these data consistently show that people with MS are more likely to be colonized by *etx*^+^ strains of *C*. *perfringens* in their gut microbiome than are age- and gender-matched people who are HCs. Our data suggest that there is likely a yet-to-be defined threshold of relative abundance of ETX-producing strains, with relative abundance beyond this threshold contributing to MS. The complex dynamic between *C*. *perfringens* type A strains and the *etx*-encoding strains is likely important, since type A strains compete with other *C*. *perfringens* strains for resources and because conjugative transfer between *etx* plasmid-harboring type D and *etx*-negative type A have been previously demonstrated in vitro (75).

### Comparison of patient-derived, laboratory, and environmental isolates.

**Figure 3:**
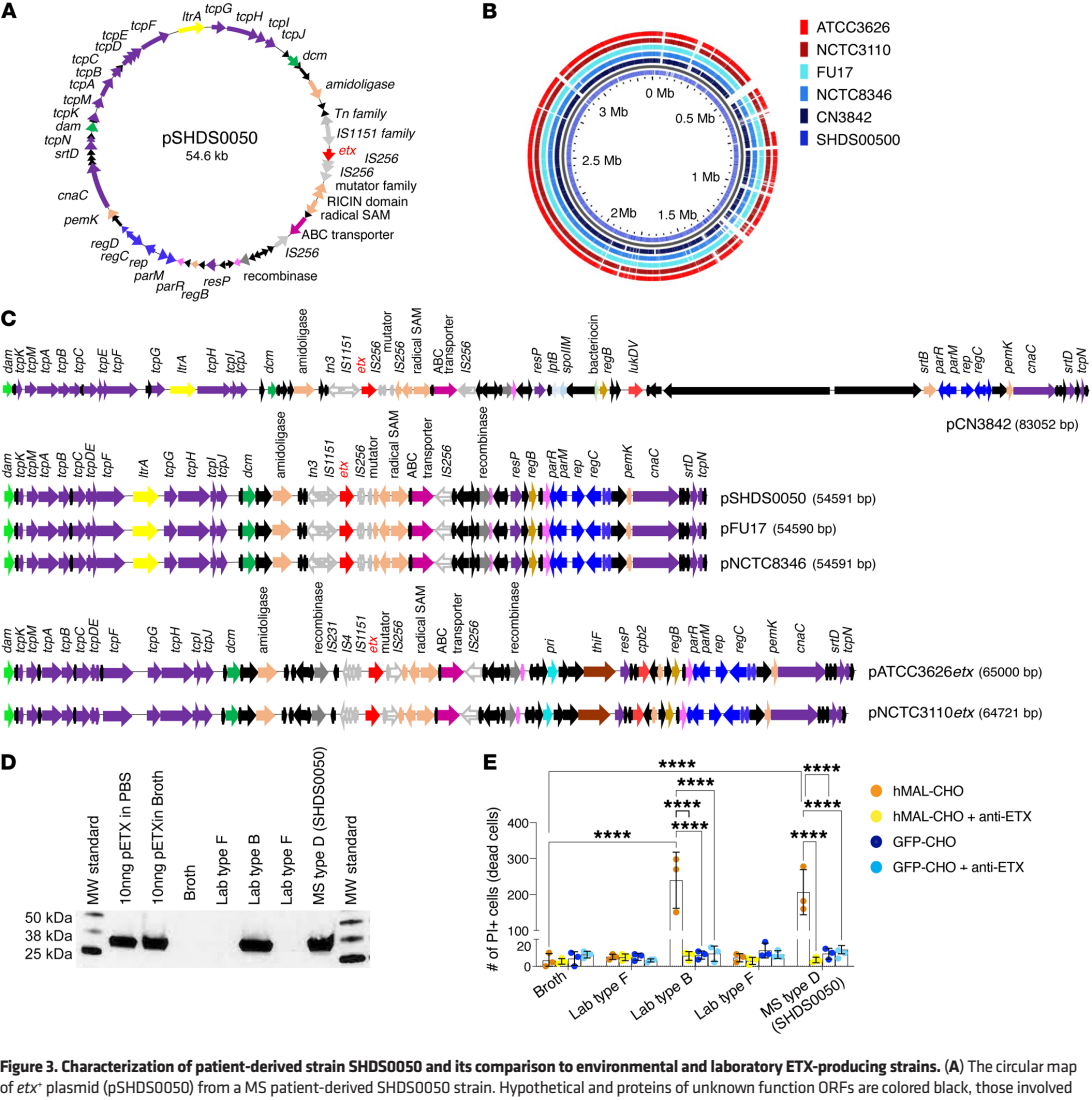
Characterization of patient-derived strain SHDS0050 and its comparison to environmental and laboratory ETX-producing strains. (**A**) The circular map of *etx*^+^ plasmid (pSHDS0050) from a MS patient-derived SHDS0050 strain. Hypothetical and proteins of unknown function ORFs are colored black, those involved in conjugation are purple, toxin ORFs are red, DNA methylases are green, transposases and recombinases are gray, ribonucleases are pink, plasmid replication ORFs are blue, conserved *etx* plasmid ORFs are red, and ABC transporters are magenta. (**B**) GView BLAST Atlas map comparing the circular chromosomes of pSHDS0050 as the reference genome to *etx*^+^strains CN3842, FU17, NCTC8346, ATCC3626, and NCTC3110. Type D chromosomes are shades of blue, while type B are shades of red. Colored regions for genomes indicate a BLAST hit present in the reference genome. Empty slots indicate no matching hits. SHDS0050 genome is distinct from other strains. (**C**) Linearized plasmid map comparisons comparing pSHDS0050 with the *etx* plasmids of 3 type D collection strains pCN3842, pFU17*etx*, and pNCTC8346*etx*, and 2 type B strains pATCC3626*etx* and pNCTC3110*etx*. The linearized maps use the same color scheme as A, with different transposase ORFs having different pattern fillings. The 3 type D strains share the same gene content and share many of the same genes. (**D**) Western blot analysis for pETX production from indicated *C*. *perfringens* strains. pETX spiked into PBS or broth were used as positive controls. Broth only was used as a negative control. (**E**) Susceptibility of hMAL-CHO or GFP-CHO cells to activated ETX. To confirm if cell death was ETX mediated, broth was pretreated with a neutralizing anti-ETX antibody. Broth alone was used as a negative control. Cell death was determined by PI staining. *n* = 3. ****P < 0.0001 determined by 2-way ANOVA with Šidák’s multiple comparisons test.

We next sought to determine if patient-derived isolates from people with MS shared features with known environmental isolates and if they produced functional ETX. We conducted whole genome sequencing of an isolate derived from a patient with MS (SHDS0050), several environmental isolates, including type D strains isolated from ruminants, and our laboratory type B strain. We successfully sequenced a closed 54.5 kb plasmid derived from isolate pSHDS0050 (Figure 3A). This plasmid contained 63 ORFs and an *etx* locus flanked by mobile genetic elements. As with other *etx* plasmids, pSHDS0050 had a Tn3 (in the opposite orientation) and an IS*1151* transposase upstream of *etx* (76), but had 2 IS*256* and a mutator type transposases directly after (Figure 3C). The plasmid resembled other highly conjugative plasmids of the pCW3 family that are found in *C*. *perfringens* strains, as it contained the *tcp* locus and other genes associated with conjugation (77) and the central control region containing the *parMRC* partitioning system (78). The plasmid lacked other toxin genes such as *cpe* and *cpb*. When comparing the type D plasmid derived from a patient with MS to the other *etx*-containing type B and D plasmids sequenced in this study, pSHDS0050 maintained the same conserved genes such as *regB*, *pemK*, *amidoligase*, *permease*, RICIN domain containing genes, and a radical *SAM* gene (Figure 3C). Interestingly, pSHDS0050 had identical plasmid architecture to the sheep isolate NCTC8346 and the goat isolate FU17 (Figure 3C), which suggests that ruminants may be a potential vector for infecting humans, as originally hypothesized by Murrell (79). Although sharing much of the same genomic content, these type D plasmids were neither as large as the 64.7 kb *etx* plasmids sequenced in this study from the type B strains ATCC3626 and NCTC3110 nor the published type B strain, NCTC8533 (Figure 3C) (80). pSHDS0050 lacked genes such as *cpb2*, *thiF* and *pri* present in those plasmids.

We next compared the circularized chromosomes of the type D strains CN3842, NCTC8346, and FU17, and the type B strains ATCC3626 and NCTC3110 to ensure that patient strain SHDS0050 was not a collection strain contamination. A GView BLAST atlas plot revealed that the patient-derived strain had multiple unique regions within its chromosome, distinguishing it from the other isolates (Figure 3B).

To evaluate production of epsilon protoxin (proETX) and pathogenicity, we cultured SHDS0050, the ATCC type B strain, and a ATCC type F strain in TGY broth under anaerobic conditions and assessed supernatants for proETX by Western blot. The strain derived from a patient with MS produced an approximately 32.9 kDa proETX protein of similar mobility to the ATCC type B strain (Figure 3D). The ATCC type F strain was negative for ETX production, as anticipated. To evaluate cytotoxicity, harvested supernatants were treated with trypsin to activate ETX. Next, CHO cells expressing the human ETX receptor, MAL (hMAL-CHO) (54), or control CHO cells expressing GFP (GFP-CHO) were treated with trypsin-activated supernatants. Cell death was determined by propidium iodide (PI) exclusion assay. To confirm that the cytotoxic effects were ETX mediated, trypsin-activated supernatants were also treated with a neutralizing anti-ETX antibody (81) prior to CHO cell treatment. Only the trypsin-activated supernatants from the lab type B and type D strain derived from a patient with MS, SHDS0050, induced hMAL-CHO cell death (Figure 3E). Cell death was not observed in hMAL-CHO cells treated with broth alone, and GFP-CHO cells were insensitive to all treatment conditions (Figure 3E). Anti-ETX antibody treatment inhibited hMAL-CHO cell death, indicating that the supernatant cytotoxicity was ETX mediated. Collectively, these data indicate that our *C*. *perfringens* type D strain derived from a patient with MS possessed typical type D plasmid architecture, was competent to produce functional ETX, and conceivably could have arisen from a ruminant source.

SNPs in bacterial genes have been linked to microbial fitness (82) and the ability of pathogens to cause disease (83). Large-scale genomic analyses have identified abundant SNPs in the *C*. *perfringens* genome (84, 85). To characterize these variants, we collated 17 *etx* genes with coding DNA sequences (CDS) available through the National Center for Biotechnology Information (NCBI) database and performed a comparative alignment analysis. The SNP at site 762 carried the least conserved SNP among the 6 that we identified and was the only SNP present in the coding sequence of activated ETX. The minor allele at the 762 site was a substitution of guanine for adenine (762G), regardless of C. perfringens toxinotype (Supplemental Figure 5C).

The *etx* genes isolated from the human fecal samples in our study predominantly (95%, 20 of 21) carried the minor allele at site 762, 762G (Supplemental Figure 5D). This variant resulted in a synonymous mutation at residue 254, where Serine corresponded to residue 222 in activated ETX, in the receptor binding domain (49). Emerging evidence indicates that synonymous mutations, previously assumed to be phenotypically neutral, contribute to microbial fitness. Furthermore, synonymous mutations can affect mRNA secondary structure, protein translation, and protein folding (86).

### ETX overcomes CNS immune privilege.

Circulating myelin-autoreactive lymphocytes are common in the general population (87). Despite the prevalence of myelin autoreactive lymphocytes among humans, widespread autoimmunity is minimized by mechanisms of peripheral tolerance and CNS barriers to preserve immune privilege (88–90). The importance of CNS barriers in maintaining immune privilege is well demonstrated in active immunization models of EAE. In this model system, animals are immunized with a myelin antigen, typically a myelin-specific protein or peptide, in complete Freund’s adjuvant (CFA) (91). In most paradigms, immunized animals do not develop clinical or histologic CNS disease unless they also receive PTX, which targets CNS endothelial cells at the BBB (91–93). Similarly, in many myelin peptide-specific T cell receptor (TCR) transgenic models, EAE occurs infrequently unless animals receive PTX, despite the TCR repertoire being biased toward unusually high frequencies of autoreactive clones (91, 94). Although PTX is extensively used in EAE to overcome immune privilege, it is clinically irrelevant to MS.

Knowing that bloodborne ETX specifically targets CNS endothelial cells (26), we wanted to test if ETX was sufficient to breach CNS immune privilege in the presence of circulating myelin autoreactive lymphocytes. We adapted a widely used immunization model of EAE using the immunodominant peptide in the myelin oligodendrocyte glycoprotein (MOG) (91). On day 0, female C57BL/6 mice received an s.c. injection of 200 μg of synthesized mouse MOG_35-55_, emulsified in 50 μl of CFA. On days 0 and 2, mice received either PTX at 5 μg/kg body weight (approximately 100 ng per mouse), ETX at 50 ng/kg body weight (approximately 1 ng per mouse), or ETX at 500 ng/kg body weight (approximately 10 ng per mouse), all delivered i.p.

**Figure 4:**
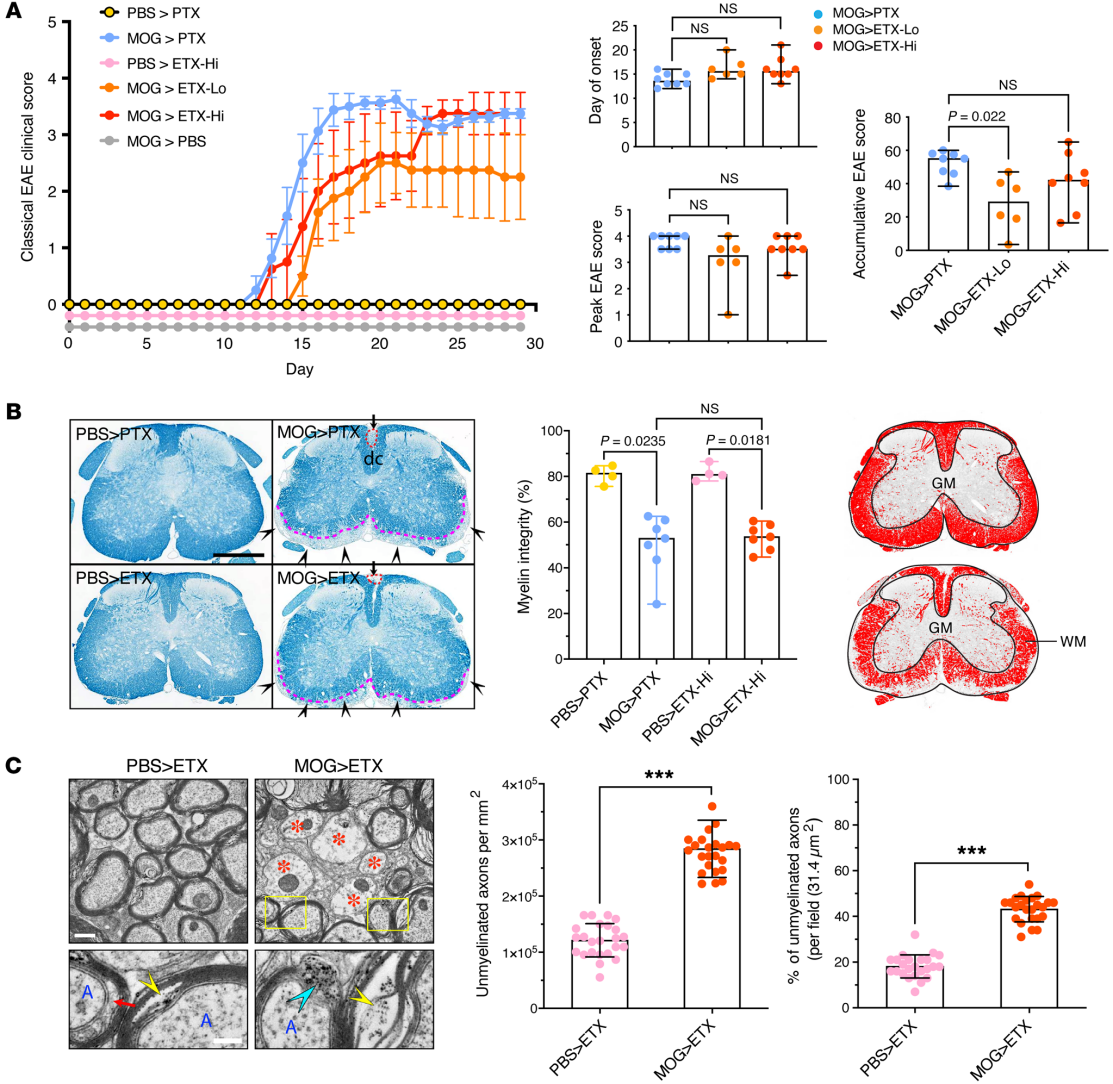
Comparison of ETX- and PTX-EAE in the spinal cord using the classical scoring scale. (**A**) Classical EAE time-course (left panel) of mice from indicated experimental groups. Mice immunized with CFA/PBS (PBS) or CFA/MOG_35-55_ (MOG) were injected i.p. with PTX at 5 μg/kg, ETX at 50 ng/kg (ETX-Lo), or ETX at 500 ng/kg body weight (ETX-Hi) on days 0 and 2 following immunization. Quantification of day of disease onset, peak EAE score, and increasing EAE score (right panels). (**B**) Representative sections of lumbar spinal cord from mice sacrificed at day 30 stained with Luxol Fast Blue (myelin stains blue). Red circles and black arrowheads indicate demyelination in the dorsal column (dc) or in the lateral and ventral funiculi, respectively. Dashed pink lines indicate lesion border in ventral white matter (WM) tracts. Quantification of myelin integrity (%, middle panel) generated from binary images (right panel). (**C**) Electron microscopy (left panel) and quantification (middle and right panels) evaluating demyelination in ETX-EAE mice. Red asterisks indicate demyelinated axons; yellow rectangles highlight ultrastructural changes of myelin sheath; cyan arrowhead indicate decompacted myelin sheath; yellow arrowheads indicate splitting myelin sheath and debris; red arrow indicates expanded space between an axon and myelin. Data in left panel of A are mean ± SEM; data in right panels of **A** and **B** represent median ± range; Kruscal-Wallis test (nonparametric); NS, not significant; *n* = 4 mice for PBS > PTX and PBS > ETX-Hi controls, *n* = 6 mice for MOG > EAE-Lo, and *n* = 8 mice groups MOG > PTX and MOG > ETX-Hi. Data in **C** represent mean ± SD; unpaired *t* test; *** *P* < 0.001; *n* = 24 fields from 2 mice. Scale bars: 1 mm (**B**), 500 nm (**C**, top panels), and 200 nm (**C**, lower panels). Similar results for 2 independent experiments.

ETX, like PTX, was sufficient to induce clinical disease in mice immunized with MOG peptide 35–55 (MOG_35-55_) (Figure 4A). In the absence of either toxin, MOG_35-55_–immunized animals remained healthy and without an observable phenotype. Disease activity induced by ETX occurred at doses significantly lower than that of PTX (50 ng/kg or 500 ng/kg for ETX, and 5 μg/kg for PTX). Onset, temporal course, and peak clinical deficits were similar for ETX- and PTX-induced EAE when using the classical EAE scoring scale (Figure 4A). The magnitude of demyelination in the spinal cord and the ultrastructural characteristics of demyelination were similar comparing ETX-induced EAE (ETX-EAE) with PTX-induced EAE (PTX-EAE, Figure 4, B and C). These results indicate that ETX is a potent inducer of EAE in the context of a MOG_35-55_/CFA immunization paradigm.

### ETX-EAE induces multi-focal demyelination.

**Figure 5:**
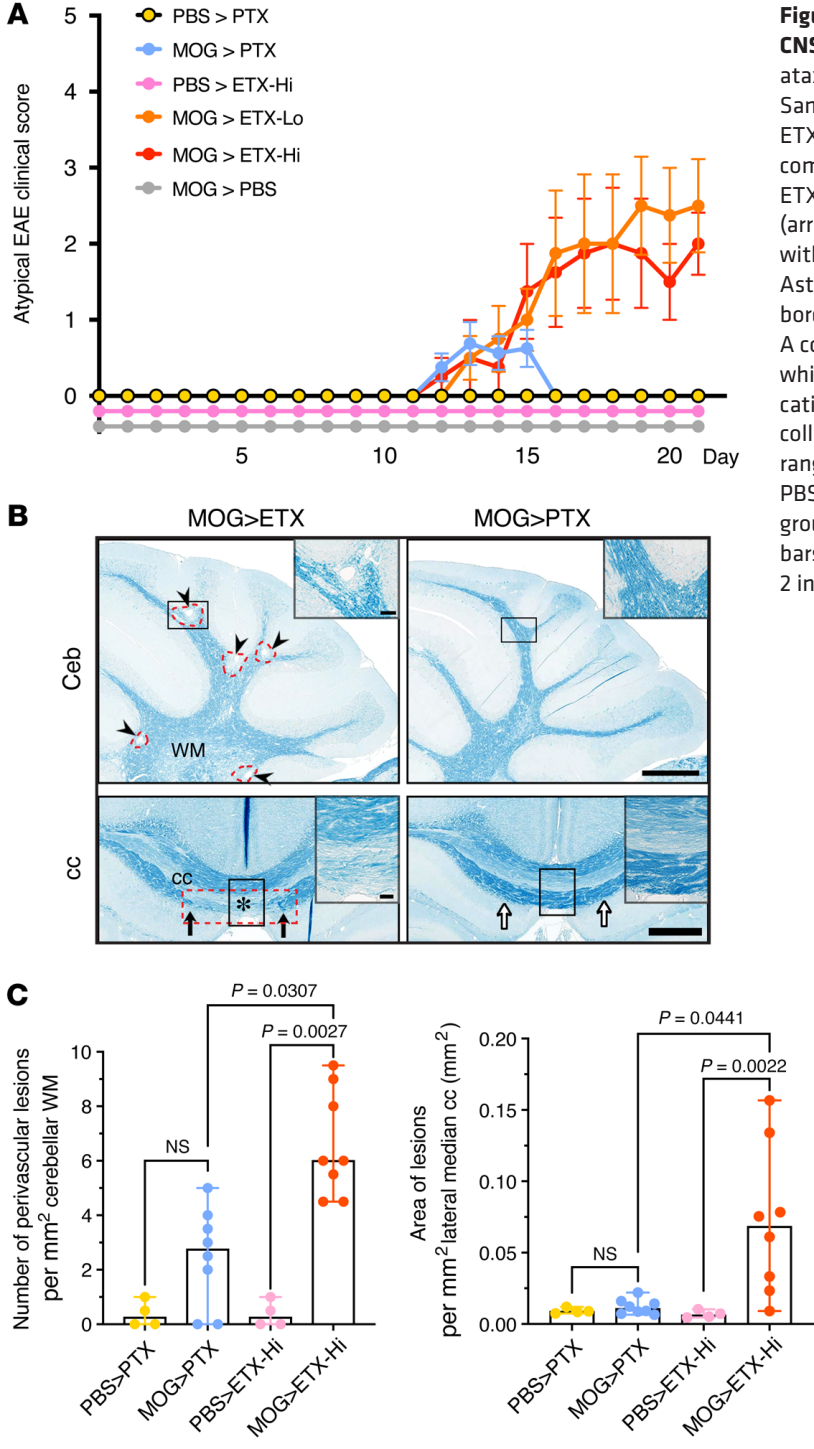
ETX-EAE is characterized by multifocal demyelination in the CNS. (**A**) ETX-EAE mice developed atypical EAE, which is characterized by ataxia, along with classical EAE symptoms defined by ascending paralysis. Same treatment conditions as in Figure 4, CFA/MOG_35-55_ followed by ETX (ETX-EAE) or PTX (PTX-EAE). (**B**) ETX targets broader brain regions compared with PTX including cerebellum (Ceb) and corpus callosum (cc). ETX-EAE mice (left column) exhibit more focal demyelinating lesions (arrowheads and red dashed circles) in the cerebellum and corpus callosum within WM tracts when compared with PTX-EAE mice (right column). Asterisk in lower row indicates demyelinated cc. Black arrows indicate borders of a cc lesion, which is also framed with a red dashed rectangle. A corresponding location in PTX- EAE mice (right column) is indicated by white arrows. Regions framed with black boxes are shown at high magnification in inserts. (**C**) Quantification of lesions in the cerebellum and corpus collosum. Data in **A** are mean ± SEM, and data in **C** represent median ± range; Kruscal-Wallis test (nonparametric). *n* = 4 mice for PBS > PTX and PBS > ETX-Hi controls, *n* = 6 mice for MOG > ETX-Lo, and *n* = 8 mice for groups including MOG > PTX and MOG > ETX-Hi. NS, not significant. Scale bars: 1 mm in **B** and 100 μm in the inserts. Similar results were achieved in 2 independently repeated experiments.

ETX-EAE groups displayed a wider array of behavioral deficits when compared with PTX-EAE, including ataxia, head tilt, imbalance, axial rotation, and left/right leaning, as captured by the atypical EAE scoring scale (Figure 5A and Supplemental Figure 6). Demyelination in PTX-EAE mice was largely restricted to the spinal cord (Figures 4 and 5). In contrast, ETX-EAE mice developed multifocal demyelination more typical of MS (Figure 5B). Compared with PTX-EAE, ETX-EAE mice had nearly twice as many lesions in the cerebellum, and lesions in the corpus callosum were only observed in the ETX-EAE group (Figure 5C). ETX-induced EAE showed perivenular cuffs of mononuclear cells and mononuclear infiltrates that correlated with demyelination (Supplemental Figure 7).

**Figure 6:**
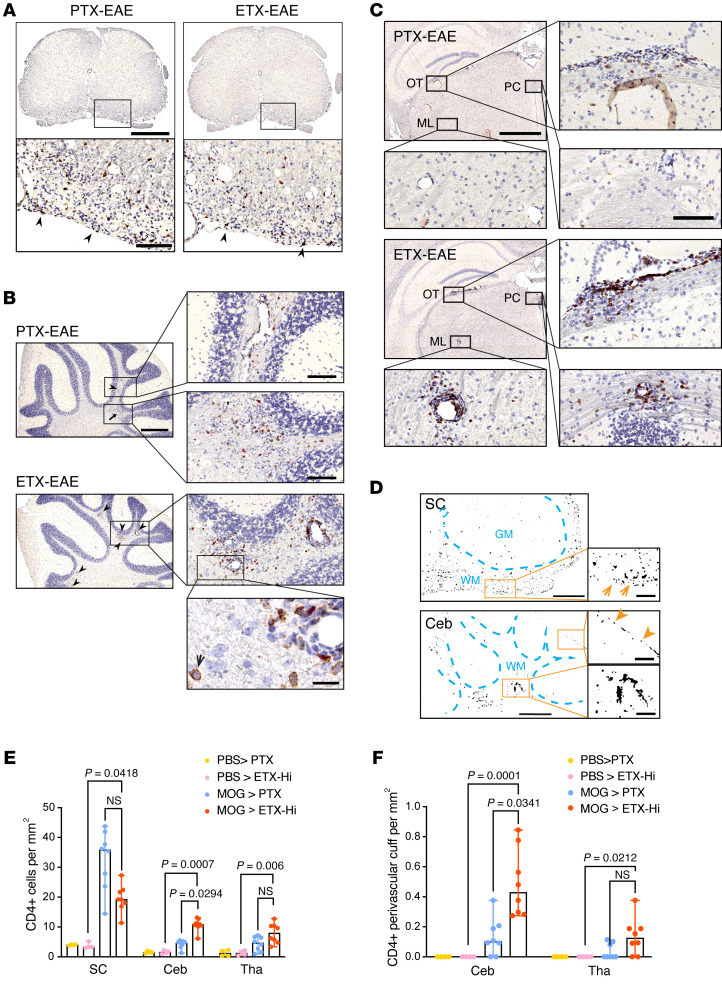
ETX-EAE mice show increased CD4^+^ lymphocyte infiltration in the cerebellum and thalamus compared with PTX-EAE. Sections from mice sacrificed at day 30 after immunization with CFA/MOG_35-55_ followed by ETX (ETX-EAE) or PTX (PTX-EAE) injection were immunostained with anti-CD4 antibody (brown) and counter-stained with hematoxylin (purple). (**A**–**C**) Representative CD4 staining in spinal cord (**A**), cerebellum (**B**) and thalamus (**C**). In ETX-EAE mice, CD4^+^ cells were found in perivascular cuffs and surrounding parenchyma indicated by arrowheads and shown in boxed regions of **B** and **C**. CD4^+^ cells from PTX-EAE mice were localized to perivascular space or scattered (arrowhead and arrow). Boxed regions shown at higher magnifications to illustrate morphological details. A high magnification micrograph (bottom panel in **B**) depicts CD4^+^ staining (black arrow). OT, optic tracts; ML, medial lemniscus; PC, posterior commissure. (**D**) ImageJ-generated binary drawings on CD4^+^ distribution in the white matter (WM) of a PTX-EAE spinal cord (SC, orange arrows) and ETX-EAE cerebellum (Ceb). In Ceb, orange arrowheads indicate CD4^+^ cells confined to a single layer in the meninges (upper boxed region). Perivascular localization of CD4^+^ cells shown at a higher magnification in the cerebellum of ETX-EAE mice (lower boxed region). GM, gray matter. Scale bars: 1 mm (top row **A**, and left column **B**), 2 mm (top and third rows in left column in **C**), 200 μm (high magnification of boxed regions **A**–**C**), 500 μm (**D**, left column), 100 μm (**D**, right column), and 50 μm (**B**, bottom right panel). (**E** and **F**) Quantification of number of CD4^+^ cells (**E**) and CD4^+^ perivascular cuffs (**F**) in indicated CNS regions. Data represent median ± range; Kruscal-Wallis test (nonparametric). NS, not significant. *n* = 4 and 8 mice for controls (PBS > PTX; PBS > ETX-Hi) and EAE groups (MOG > PTX; MOG > ETX-Hi), respectively. Similar results were obtained from 2 independent experiments.

**Figure 7:**
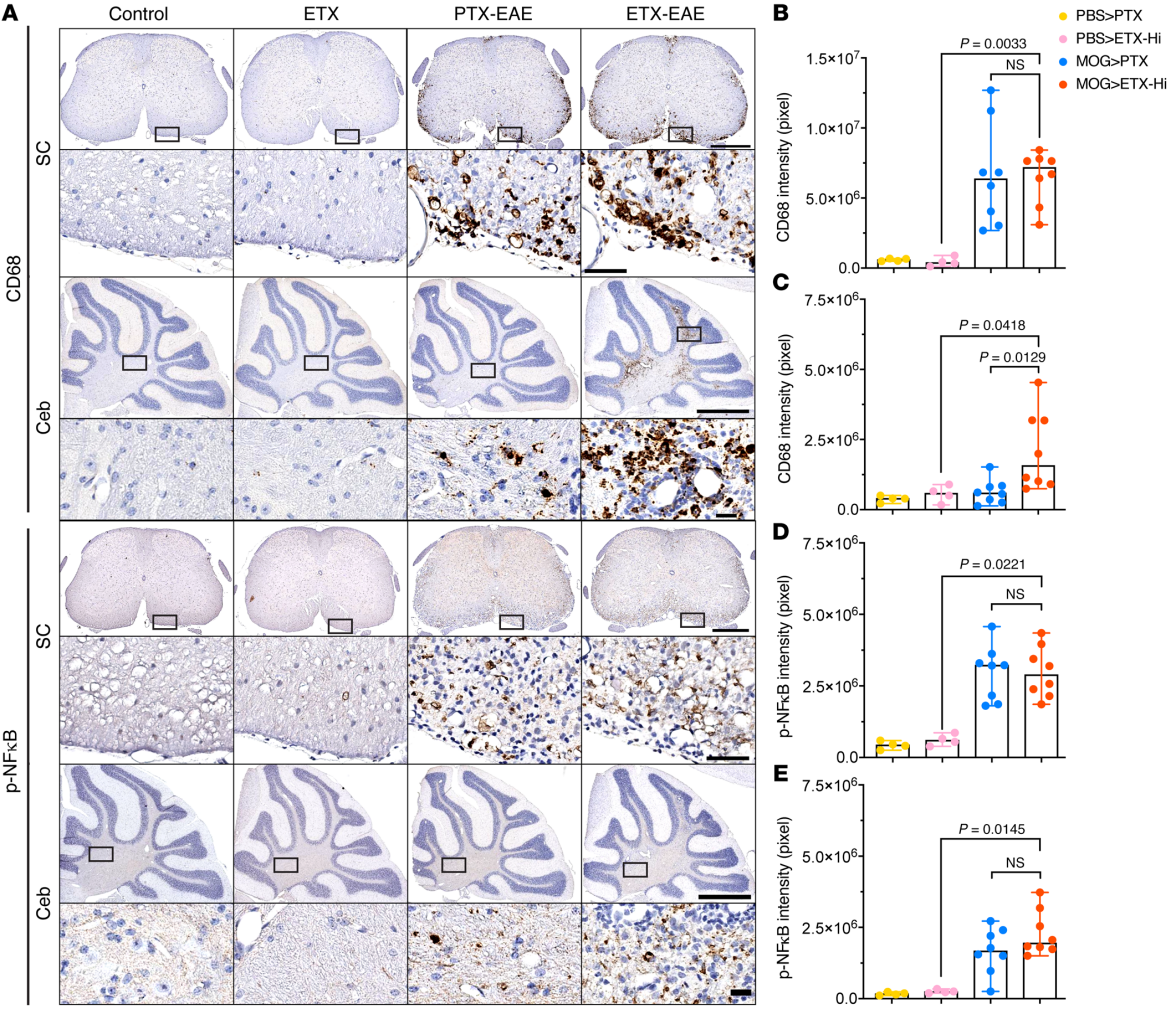
ETX-EAE mice show increased CD68^+^ microglia in the cerebellum of ETX-EAE mice compared with PTX-EAE mice, despite similar activation in the spinal cord and a comparable profile of transcription factor NFκB p65 in both models. (**A**) Representative sections from mice sacrificed at day 30 after immunization with CFA/MOG_35-55_ and followed by either ETX (ETX-EAE) or PTX (PTX-EAE) injections were immunostained with anti-CD68 antibody or anti-phospho-NFκB p65 (Ser 276). A rectangle-framed region in each condition in **A** is shown at a higher magnification beneath the corresponding section. SC, spinal cord; Ceb, cerebellum. Note that adjacent sections in each group were used for staining against CD68 and phospho-NFκB p65. Scale bars: 500 μm (bottom, spinal cord); 50 μm (top, spinal cord); 1 mm (bottom, cerebellum); 20 μm (top, cerebellum). (**B**–**E**) Statistical analysis of staining intensity for CD68 (**A** and **B**) and phosphor-NFκB p65 (pNFκB; **D** and **E**) in the spinal cord (**B** and **D**) and cerebellum (**C** and **E**), respectively. Data represent median range; Kruscal-Wallis test (nonparametric). NS, not significant. *n* = 4 and 8 mice for controls (Control: PBS > PTX; ETX: PBS > ETX-Hi) and EAE groups (PTX-EAE: MOG > PTX; ETX-EAE: MOG > ETX-Hi), respectively.

We next wanted to compare the immune infiltrates in brain and spinal cord between the ETX- and PTX-EAE models. We examined histologic sections in ETX-EAE, PTX-EAE, and controls for infiltrating CD4^+^ lymphocytes. While both models induced similar demyelination in the spinal cord, PTX-EAE showed significantly more infiltrating CD4^+^ T cells in the spinal cord compared with ETX (Figure 6, A and E). For the cerebellum, there were significantly more infiltrating CD4^+^ lymphocytes and more CD4^+^ lymphocytes in perivascular cuffs in the ETX-EAE model compared with PTX-EAE (Figure 6, B–F). In the thalamus, there was a similar trend toward more peri-venular CD4^+^ T cell infiltrates in ETX-EAE when compared with PTX-EAE (Figure 6, C–F). In the spinal cord, similar activation of inflammatory processes was observed in both models at peak disease, based on immunohistochemical analysis of phospho-NFκB p65, CD68, and CD45 (Figure 7 and Supplemental Figure 8). In the cerebellum, however, CD68 was significantly increased in ETX-EAE but not PTX-EAE mice (Figure 7, A–C). A similar trend was also observed in the cerebellum for phospho-NFκB p65 and CD45, but this did not achieve statistical significance (Figure 7, A–E and Supplemental Figure 8). These results, collectively with data from Figures 4 and 5, indicate that ETX induced multifocal, inflammatory demyelination in a neuroanatomic distribution more consistent with MS and with a stronger correlation between immune infiltrates and demyelination in the ETX-EAE model compared with PTX-EAE (Supplemental Figure 9).

Human lymphocytes, but not mouse lymphocytes, express the ETX receptor, MAL (54, 58, 95). While it seemed unlikely that ETX functions to induce active EAE by affecting peripheral immunity, we nevertheless wanted to test this possibility through tracking MOG_35-55_–specific T cell cytokine responses via antigen recall experiments. To track the fate of MOG_35-55_–specific T cell responses in vivo (96–98), we transferred naive, purified MOG_35-55_–specific CD4^+^ T cells from MOG_35-55_–specific TCR transgenic (2D2) mice into recipient WT B6 mice 24 hours before induction of active EAE by s.c. immunization with MOG_35-55_ in CFA. Immunized mice then received either PBS, 10 μg/kg PTX, or 500 ng/kg ETX immediately after immunization and again 48 hours later (Supplemental Figure 10A). We monitored mice for the onset of clinical disease (Supplemental Figure 10B), and, 14 days following immunization, we generated single-cell suspensions from inguinal lymph nodes, cervical lymph nodes, and from the CNS (brain and spinal cord) and processed these for either direct analysis of basal cytokine production by flow cytometry (Supplemental Figure 10C) or for cytokine production after antigen recall via ex vivo restimulation with MOG_35-55_ for 72 hours (Supplemental Figure 10D). For both sets of experiments we assessed intracellular cytokine production by flow cytometry. MOG_35-55_–specific 2D2 were positively defined as CD45^+^, CD3/5^+^, CD4^+^, and Thy1.1^+^. Frequencies of TNF-α, IFNγ, or GM-CSF positive CD4^+^ T cells did not significantly differ between PBS controls and ETX-treatment in cervical or inguinal lymph nodes, except for a small difference in the frequency of TNF-α^+^ T cells from inguinal lymph nodes, following 72 hours of restimulation with MOG_35-55_ (Supplemental Figure 10D). In CNS tissue, there were no differences noted in the frequencies of TNF-α, IFNγ, or GM-CSF positive CD4^+^ T cells between the ETX and PTX treatment groups. These results support the conclusion that ETX was not functioning through significant effects on peripheral immunity and did not affect MOG_35-55_–specific T cell responses during the induction of active EAE.

### ETX and PTX alter the CNS endothelial cell transcriptome to induce genes involved in BBB dysfunction.

To gain insight into the functional mechanisms of ETX and PTX in overcoming immune privilege at the CNS endothelial barrier, we conducted bulk RNA-Seq on CNS endothelial cells isolated from animals treated with ETX, PTX, or PBS and compared transcriptional profiles.

**Figure 8:**
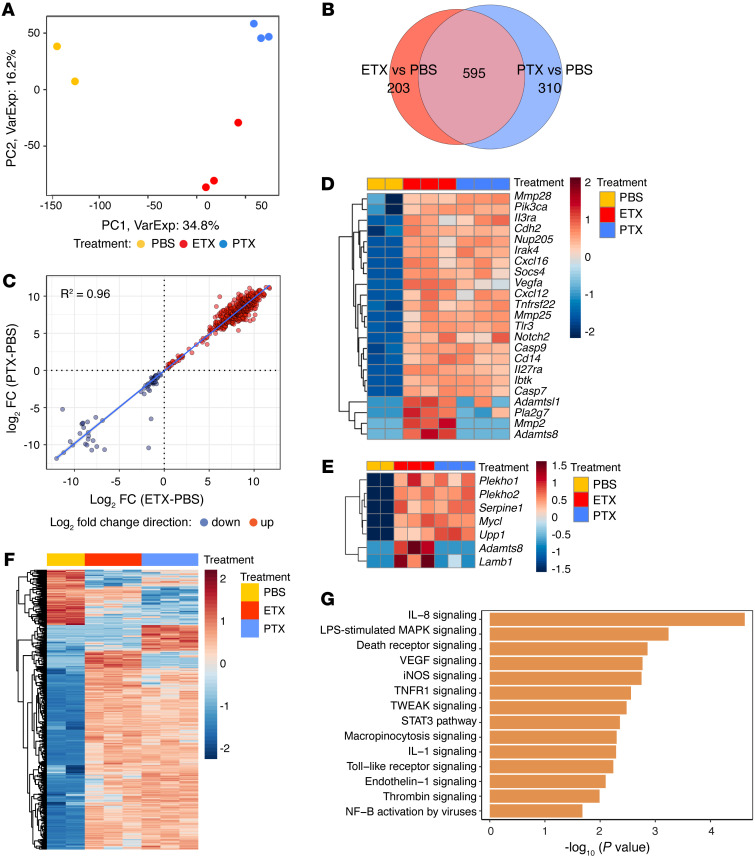
Transcriptome analysis of ETX- and PTX-treated CNS endothelial cells from spinal cord. (**A**) Principal-component (PC) analysis of RNA-Seq data, based on the top 1,000 most variable genes. Each symbol represents a biological replicate, and each component is indicated with the amount of variation that it explains. *n* = 3 mice for PTX and ETX treatment and *n* = 2 mice for PBS treatment. (**B**) Venn diagram showing extent of overlap between genes differentially expressed in ETX- or PTX-treated samples relative to control (PBS). (**C**) Correlation analysis of log_2_ fold-changes between genes that change in both ETX and PTX displayed as a scatterplot, R^2^ = 0.96. (**D**) Heat map of DEGs of interest relevant to immune privilege (FDR < 0.10) in ETX or PTX. (**E**) Heat map of DEGs induced by ETX and/or PTX reported by Munji (101) in a BBB dysfunction module. (**F**) Heat map of all DEGs induced by ETX and/or PTX relative to PBS control. (**G**) Selected pathways predicted to be activated in ETX treated cells by Ingenuity Pathway Analysis (*P* < 0.05 and *z* score ≥ 1) based on the differentially expressed genes compared with control cells.

Mice were treated with PBS, ETX (500 ng/kg BW), or PTX (5 μg/kg BW) on 2 consecutive days followed by isolation of CNS endothelial cells from spinal cord for bulk RNA-Seq. CNS endothelial cells were isolated from spinal cords, as previously described (99), 16 hours after the second toxin dose. Bulk RNA-Seq was performed and analyzed using the limma-voom workflow (100). Principal-component (PC)analysis (Figure 8A) revealed separation by treatment. PC1 distinguished control from both ETX and PTX, whereas PC2 distinguished ETX from PTX, indicating that the ETX and PTX transcriptomes are more like each other than PBS controls. We identified 798 differentially expressed genes (DEGs) between ETX and PBS treated samples, and 905 DEGs between PTX- and PBS-treated samples (FDR *q* < 0.10). Of these DEGs, 595 changed in response to both ETX and PTX treatments (Figure 8B). Comparing the fold changes (FCs) of the overlapping genes revealed a remarkable consistency in direction and magnitude of differential expression between ETX and PTX treatments (Figure 8C). Considering that PTX is an A-B toxin that functions through ADP-ribosylation of G-proteins and that ETX is a pore-forming toxin of the aerolysin family, the consistency in induced and suppressed genes was both intriguing and unexpected. A heat map displaying genes of interest revealed induction of protease, signal transduction, cytokine, and transcription factor genes potentially relevant to overcoming CNS immune privilege (Figure 8D). In Figure 8E, we show a heat map of genes differentially expressed by ETX and PTX that also were identified by Munji et al. as components of a core set of genes involved in BBB dysfunction (101). The overall gene induction in endothelial cells isolated form PTX- or ETX-treated mice shares a great degree of overlap (Figure 8F). Further analysis with of the RNA-Seq data identified signaling pathways preferentially activated by ETX (Figure 8G).

## Discussion

We presume the factors responsible for the formation of the initial lesions in MS are the same factors responsible for new lesion formation throughout the course of the disease. That is, environmental triggers for MS likely do not occur all at once at disease initiation, but rather, arise repeatedly throughout the course of disease. The episodic nature of MS disease activity aligns well with the ETX hypothesis. ETX production occurs when *C*. *perfringens* types B or D enter log-phase growth and, thus, is tied to increased abundance of these strains in the gut microbiome (28, 50). Brief cycles of log-phase growth followed by long periods of quiescence would account for episodic ETX production. MS disease activity is linked to an increased relative abundance of Bacillota (Firmicutes), which includes the genus *Clostridium*, suggesting that the MS gut microbiome episodically favors growth of this phylum (11). In addition to our findings, it is notable that the species *C*. *perfringens* is the most highly associated bacteria with neuromyelitis optica, which is an immune-mediated, demyelinating disorder affecting the spinal cord and optic nerve (102, 103).

### C.

*perfringens* type D is responsible for enterotoxemia in sheep, goats, and, less frequently, in cattle (104). The disease has worldwide distribution and can be acute, sub-acute, chronic, or fatal (105, 106). Although enterotoxemia type D is an infectious disease that can occur in the form of small outbreaks via the oral-fecal route, it is not typically a contagious disease. A variable number of ruminants harbor *C*. *perfringens* type D in their small intestine, but microorganism numbers are generally small and clinical disease does not occur unless the intestinal microbial balance is disrupted (106, 107). When large quantities of readily fermentable carbohydrates are fed to these animals, undigested starch passes into the small intestine and provides a substrate for *C*. *perfringens* to proliferate rapidly. This is followed by production of large amounts of ETX, which is then absorbed into the systemic circulation. The bacteria are passed by feces into the environment and can survive for several months in the soil. Neonates and older animals become infected via the fecal-oral route.

The Epstein Barr Virus (EBV) has been proposed as an environmental trigger for MS (108), potentially functioning through molecular mimicry (109). In humans, EBV is significantly associated with MS, but seroconversion is itself not sufficient to induce MS; approximately 94% of the general population is seropositive by age 24, yet MS risk remains relatively low (108, 110, 111). This suggests that, while EBV may play a role in MS pathogenesis, an additional, but less widely distributed causal factor, as discussed by Carl Nathan (112), is required, which we propose to be *C*. *perfringens* type B or D.

In summary, we found a strong clinical association between a specific bacterium, its toxin, and a diagnosis of MS. In addition, we found that the abundance of ETX-producing strains of *C*. *perfringens* was significantly elevated in the gut microbiome of patients with MS. We provided a biologically plausible mechanism by which ETX functions in the multistep process of CNS autoimmunity. Ultimate proof of this hypothesis, as would be the case for any environmental factor, will require a clinical trial to neutralize ETX or to eliminate *C*. *perfringens* type B or D in the human host.

## Methods

Details on participant recruitment, reagents, and experimental methods are found in supplemental materials.

### Data and materials availability.

*C*. *perfringens* chromosome and plasmid sequences were deposited into GenBank under the following accession numbers (chromosome, plasmid for each strain): *C*. *perfringens* type D CN3842 (CP116428, CP116429), *C*. *perfringens* type D NCTC8346 (CP116430, CP116431), *C*. *perfringens* type D FU17 (CP116432, CP116433), *C*. *perfringens* type D SHDS0050 (CP116434, CP116435), *C*. *perfringens* type B NCTC3110 (CP116436, CP116437), and *C*. *perfringens* type B ATCC3626 (CP116438, CP116439).

### Statistics.

Statistical analysis was performed using GraphPad Prism (v.9; GraphPad) and Microsoft Excel (2016; Microsoft). Data of Gaussian distribution are represented as mean ± SEM or SD, as indicated. Data of non-Gaussian distribution are represented as median ± range. Unpaired 2-tailed *t* test (for Gaussian distribution) or Mann–Whitney test (for non-Gaussian distribution) were used to compare 2 data sets for statistical significance. For multiple data sets, 1-way ANOVA followed by post hoc Tukey’s multiple comparisons test (for Gaussian distribution), Kruskal–Wallis test followed by post hoc Dunn’s multiple comparisons test (for non-Gaussian distribution), or 2-way ANOVA with Šidák’s multiple comparisons test were carried out to determine statistical significance as indicated. For EAE time course analysis, nonparametric Friedman’s test followed by post hoc Dunn’s test was performed. Pearson’s correlation analysis was performed to examine whether the density of CNS infiltrating lymphocytes correlated with the extent of demyelination. One phase decay model was employed for curve fitting. Multivariable logistic regression was used to test the difference while adjusting for covariates. *P* values are stated in figures and legends.

The RNA-Seq data discussed in this publication have been deposited in NCBI’s Gene Expression Omnibus (113) and are accessible through GEO Series accession number GSE223137.

### Study approval.

The HITMS Study, protocol # 1003010940, was approved by the Weill Cornell Medicine Institutional Review Board. All study participants received, reviewed, and signed a written informed consent.

The Weill Cornell Medicine IACUC approved all experimental procedures with animals, which were also consistent with the ethical guidelines of the NIH.

## Author contributions

YM conceptualized, designed, and carried out EAE experiments and performed histological and ultrastructural analyses. He conceptualized quantification methods and carried out the analyses of fecal samples by standard and TaqMan real-time PCR. He also wrote the IACUC protocol and edited the manuscript. DS conceptualized, designed, and carried out the purification of bacteria from fecal samples using the Nycodenz method, performed the genomic assembly of the human *C*. *perfringens* isolates, and created the plasmid maps for the patient and environmental isolates, as well as having edited the manuscript. JRL generated the antibodies used in the detection of ETX and assisted with the design of the study and analysis of *etx* detection in fecal samples from MS and HC study participants. She also assisted with obtaining the MS participant isolate, conducted the analysis of CHO cell cytotoxicity, isolated endothelial cells and purified RNA from mice used in the analysis of differential gene expression, assisted with writing the IRB and IACUC protocols, and edited the manuscript. SH assisted in the analysis of fecal samples and purification of isolates. BZ performed standard and TaqMan real-time PCR analyses of fecal samples, and assisted with daily monitoring and scoring of EAE mice in all experiments involving the model. JBG designed, conducted, and analyzed the EAE antigen recall and flow cytometry experiments. PZ conducted the analysis of endothelial cell differential gene expression, and constructed the principal component analysis plot, gene ontogeny plot, and heat maps for endothelial cell differential gene expression. FD assisted in the design, analysis, and strategy for endothelial cell differential gene expression. DB performed Ilumina shotgun sequencing and Nanopore long reads on purified bacterial isolates and edited the manuscript. CPP advised and assisted with CNS endothelial cell isolation and preparation of RNA for bulk RNA seq experiments. KT edited the manuscript. PNW performed Western blot analysis on supernatants from clinical isolates. KRR assisted with design of the study, interpretation of data, and edited the manuscript. SAG assisted with design of the study, developed the RedCap database for entering secure demographic and clinical information on recruited subjects, and edited the manuscript. VAF assisted with the design of the study, interpretation of results, and edited the manuscript. BAM assisted with the analysis and interpretation of the plasmid maps for patient derived strains and environmental strains and edited the manuscript. FAU provided the *C*. *perfringens* types B and D environmental isolates., provided background on *C*. *perfringens* type D in ruminants, and edited the manuscript. LZ recruited patients for the study and maintained the database. MM assisted with Western blot experiments and figure preparation. RR edited the manuscript. EB edited the manuscript and assisted with antigen recall experiments. DD assisted with plasmid assemblies. JP recruited patients for the study. UK recruited patients for the study. NN recruited patients for the study. ILB edited the manuscript. ZC reviewed the power calculation for the analysis of *etx* in human fecal samples, multivariable logistic regression, and reviewed all statistical methods. JZX assisted with RNAseq strategy, RNA quality check, library generation, and sequencing. DB supervised and designed the bioinformatics approach for endothelial cell differential gene expression. RD assisted with design of the transcriptional profiling experiments and interpretation of the RNA-Seq data sets and designed the strategy for isolation of CNS endothelial cells from whole animals for bulk RNA-seq. GFS conceptualized, supervised, and interpreted the EAE experiments. CEM designed the sequencing strategy for *C*. *perfringens* strains. TV conceptualized and supervised the overall study, conceptualized and designed the analysis of fecal samples for *etx* detection, conceptualized the EAE experiments, conceptualized and designed the CNS endothelial cell RNA-Seq experiments, analyzed and interpreted data from the *etx* detection, EAE, and CNS endothelial cell RNA-Seq experiments. They were also responsible for writing and adhering to the IRB and IACUC protocols, and wrote the manuscript. The order of the co–first authors was based on the time and effort devoted to this project.

## Supplementary Material

Supplemental data

Supplemental Methods

Supplemental Table 1

Supplemental Table 2

## Version 1 02/28/2023

In-Press Preview

## Version 2 05/01/2023

Electronic publication
