# Supplementary material for: Epsilon toxin–producing Clostridium perfringens colonize the multiple sclerosis gut microbiome overcoming CNS immune privilege
Source: J Clin Invest. 2023 May 1;133(9):e163239. doi: 10.1172/JCI163239 (PMC10145940; doi:10.1172/JCI163239)
Supplement: Supplemental data [file jci-133-163239-s173.pdf]

<u>Supplemental Data for</u>

Epsilon Toxin-Producing *Clostridium perfringens* Colonize the MS Gut and Epsilon Toxin Overcomes Immune Privilege

Yinghua Ma[1]*, David Sannino[1]*, Jennifer R. Linden[1]*, Sylvia Haigh[1], Baohua Zhao[1], John B. Grigg[2,3,4,5], Paul Zumbo[6,7], Friederike Dündar[6,7], Daniel Butler[7], Caterina P. Profaci[8], Kiel Telesford[1], Paige Winokur[9], Kareem Rashid Rumah[10], Susan Gauthier[11], Vincent A. Fischetti[10], Bruce A. McClane[12], Francisco A. Uzal[13], Lily Zexter[11], Michael Mazzucco[1], Richard Rudick[14], David Danko[7], Evan Balmuth[1], Nancy Nealon[11], Jai Perumal[11], Ulrike Kaunzner[11], Ilana L. Brito[15], Zhengming Chen[16], Jenny Z. Xiang[17], Doron Betel[6,7], Richard Daneman[8], Gregory F. Sonnenberg[2,3,4,5], Christopher Mason[7]□, and Timothy Vartanian[1,5,11,18]✉

SUPPLEMENTAL FIGURES AND LEGENDS

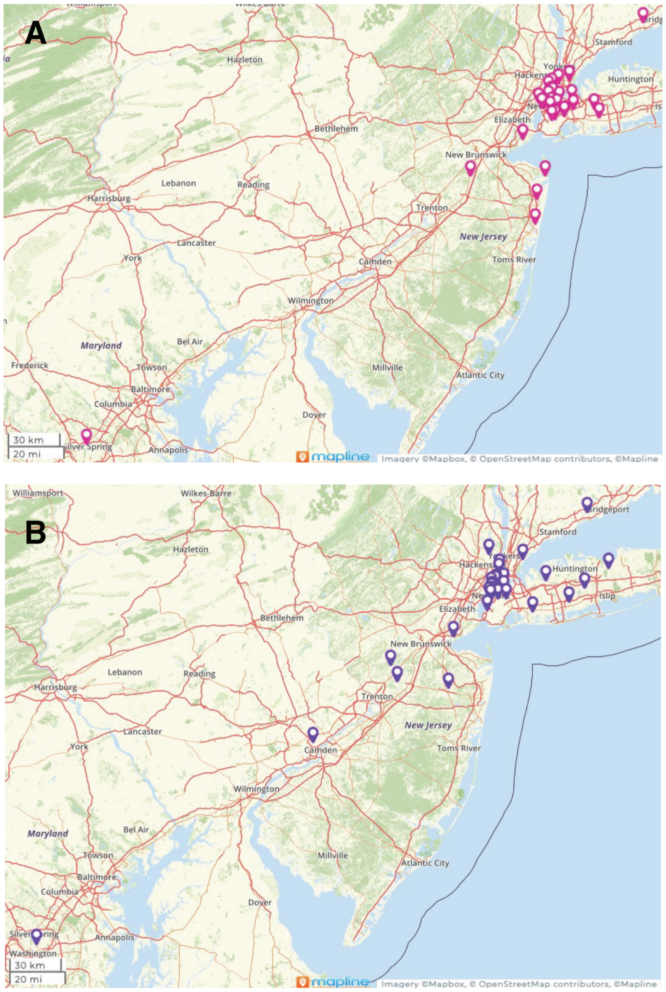

Supplemental Figure 1. Geographic location of participants in HITMS. Participants geographic locations were mapped using their home zip codes at the time of fecal sample donation. Individual participants are shown in red for MS (**A**), and purple for HC (**B**). The majority of participants in both groups, MS and HC, were from the New York metropolitan area. Each group had one participant from the Washington DC metropolitan area. The MS group had one participant from Ithaca (not shown). Maps were created using mapline (mapline.com).

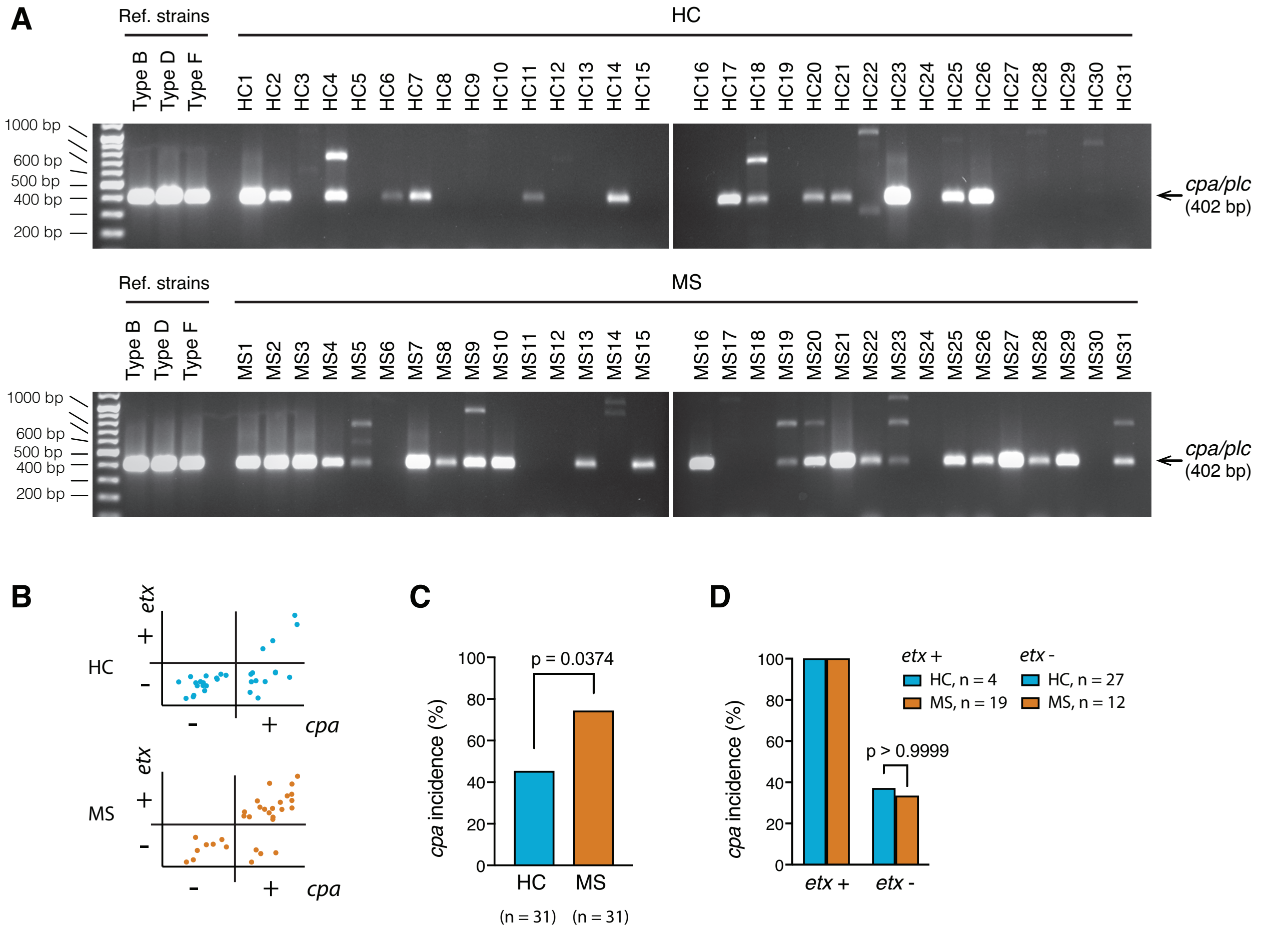

Supplemental Figure 2. ***cpa* incidence is increased in MS. (A)** PCR detection of *cpa/plc* (402 bp) from HC and MS fecal material. (**B**) Distribution of HC and MS subjects according to *etx* (y-axis) and *cpa* (x-axis) presence (+) and absence (-). (**C**-**D**) Statistical analysis of *cpa* incidence in the overall cohorts (C) and *etx*+/*etx*- subgroups (D) of HC and MS. p values determined by Fisher's exact test. Similar results were achieved in three independently repeated PCR experiments using the same subjects.

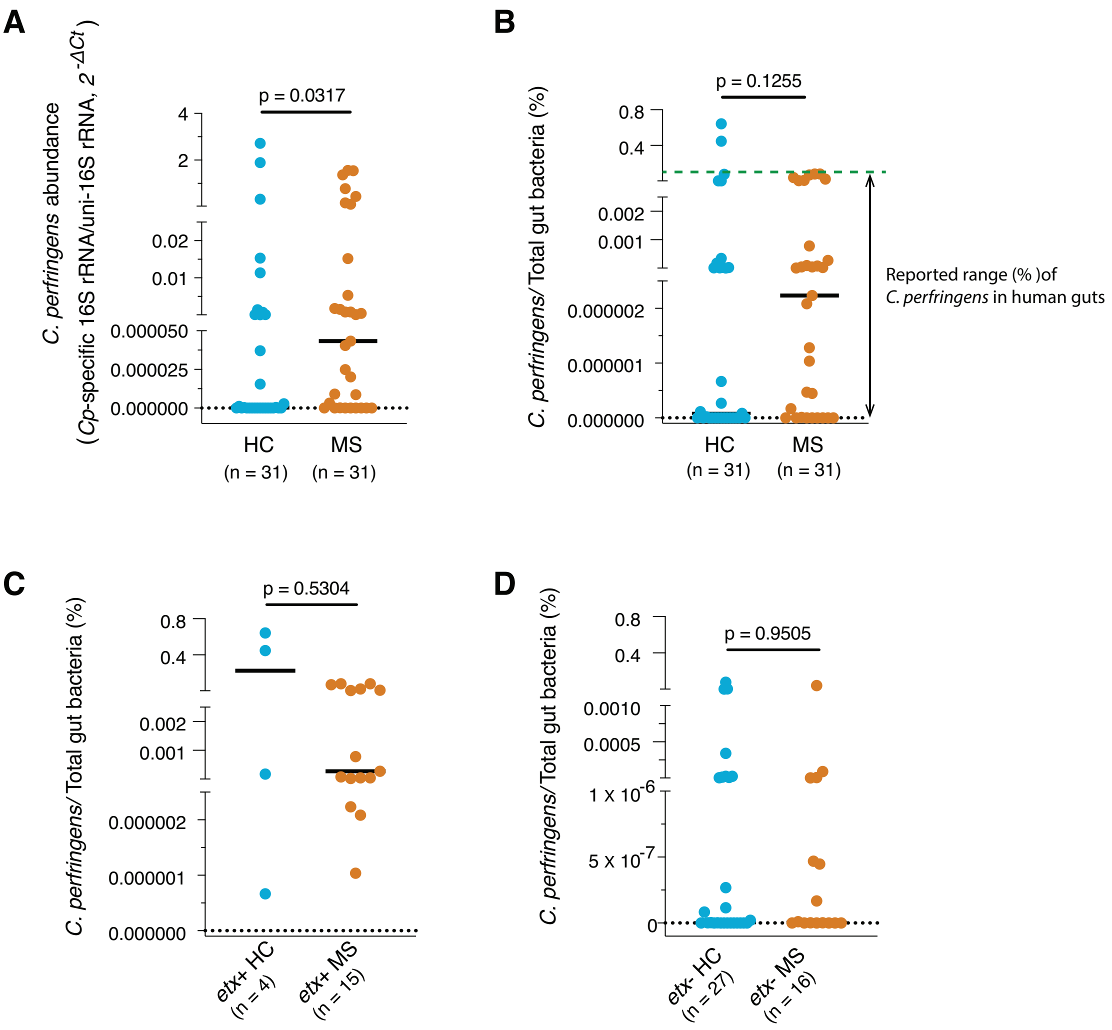

Supplemental Figure 3. ***C. perfringens* abundance is increased in MS.** (**A**) TaqMan real-time PCR analysis of *C. perfringens* abundance by simultaneously detecting *C. perfringens (Cp)*-specific and universal (Uni-) 16S rRNA genes in HC and MS participants. (**B-D**) Quantification of the percentage of total *C. perfringens* (B), or *etx*+ *C. perfringens* (C), or *etx*- *C. perfringens* (D) over total gut bacteria from HC and MS groups. The vertical line in B depicts the published range of *C. perfringens* percentage in human fecal microbiota (1, 2). Black horizontal lines indicate medians in a representative real-time PCR experiment. p value determined by Mann-Whitney test (non-Gaussian distribution). Similar results were achieved in three independently repeated experiments using the same subjects.

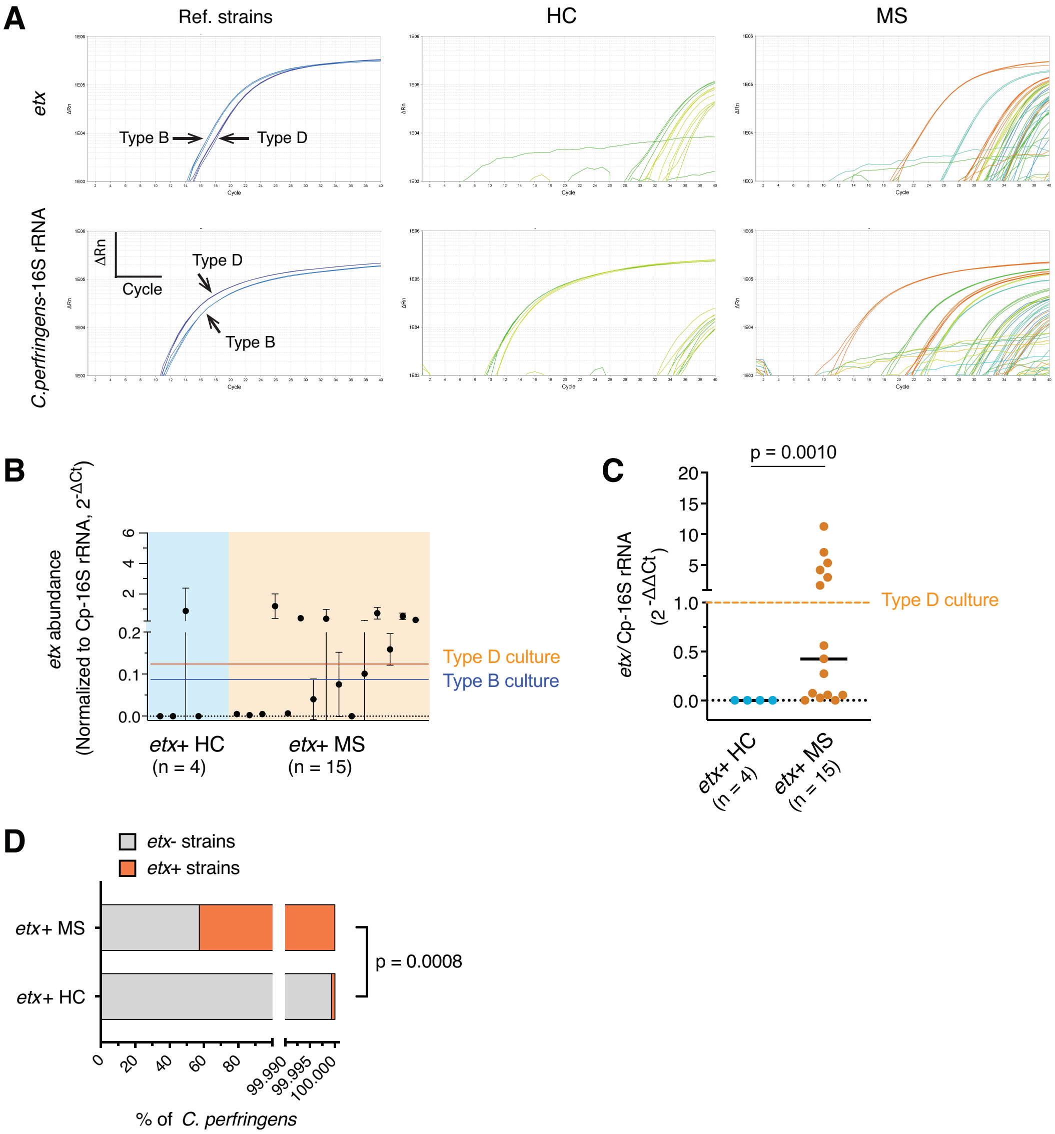

Supplemental Figure 4. **Analysis of *etx* abundance and composition of *etx*-harboring strains over *C. perfringens* communities from *etx*+ HC and MS participants**. (**A**) Representative amplification plots of simultaneous detection of *etx* and *C. perfringens* (Cp)-specific 16S rRNA by TaqMan real-time PCR. (**B**) *etx* abundance normalized to Cp-specific 16S rRNA. (**C**) $2^{-\Delta\Delta Ct}$ *analysis of etx*/Cp-16S rRNA against type D culture as calibrator. Dashed horizontal lines in B/C indicate $2^{-\Delta Ct}/2^{-\Delta\Delta Ct}$ value from culture of indicated reference strains. (**D**) Estimate of maximum

of percentages of *etx*+/*etx*- strains in *etx*+ HC and MS fecal microbiome. The estimates assume that the subjects with a $2^{-\Delta\Delta Ct}$ value above 1 (type D) contains 100% *etx*+ strains. Mann-Whitney test (non-Gaussian distribution). Data in B are mean ± SD, n = 3 technical triplicates in a representative PCR experiment. Black lines in C indicate medians. p value determined by Mann-Whitney test (non-Gaussian distribution). Similar results were achieved in three independently repeated experiments using the same subjects.

A

Position[1]	Variant		Codon change	Amino acid change
	Majority (frequency[2])	Minority (frequency[2])		
16	G (88%, 15/17)	T (12%, 2/17)	**G**TA → **T**TA	V → L
18	A (82%, 14/17)	G (18%, 3/17)	GT**A** → GT**G**	V → V
51	C (88%, 15/17)	G (12%, 2/17)	TC**C** → TC**G**	S → S
78	A (94%, 16/17)	G (6%, 1/17)	TC**A** → TC**G**	S → S
762	A (65%, 11/17)	G (35%, 6/17)	TC**A** → TC**G**	S → S
962	C (94%, 16/17)	A (6%, 1/17)	T**C**T → T**A**T	S → Y

[1] Refers to coding DNA sequence (CDS).
[2] Frequency is described by % followed by counts out of sequences collated in this study.

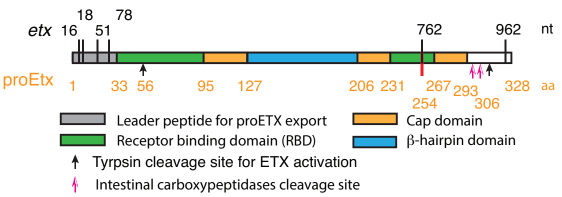

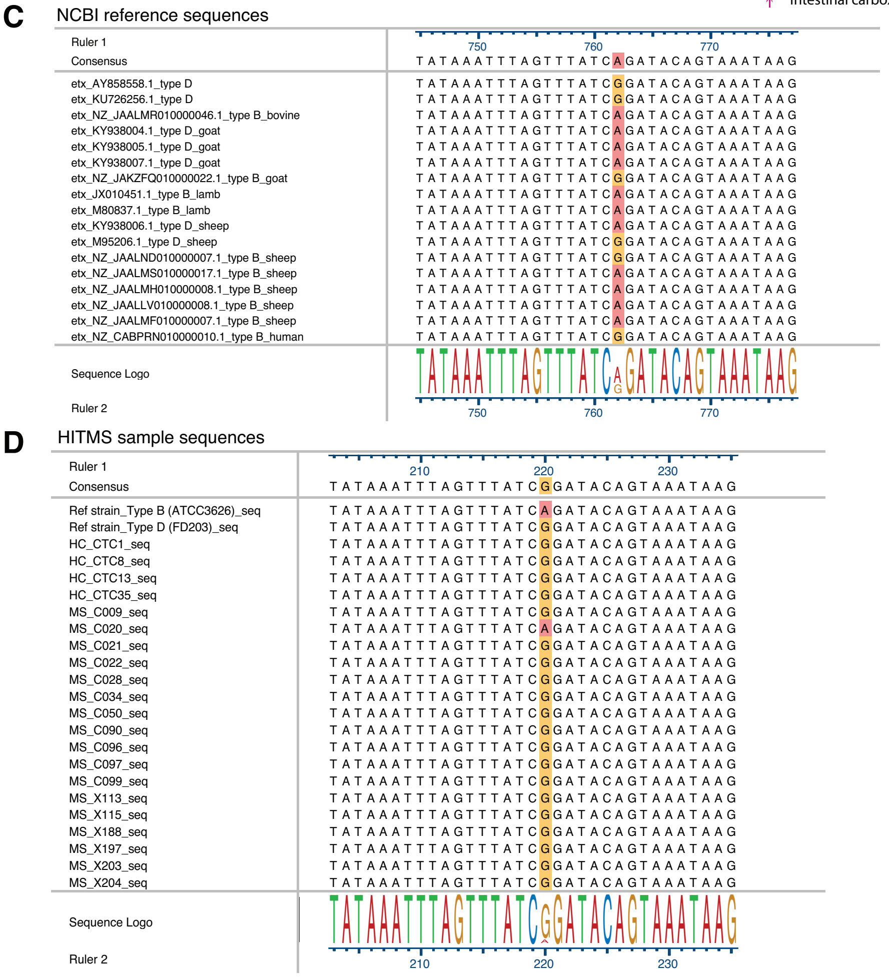

Supplemental Figure 5. ***etx* isolated from humans predominantly carries a minor SNP variant in the receptor binding domain that results in a synonymous mutation.** Reference sequences were from organisms isolated from ruminant animals with one notable exception – a recently annotated sequence from whole genome sequencing of human fecal samples (Nucleotide [https://www.ncbi.nlm.nih.gov/nuccore/1754238626]. Bethesda (MD): National Library of

Medicine (US), National Center for Biotechnology Information; [1988]. Accession No. NZ_CABPRN010000010.1]. Our analysis identified 6 SNP sites in the *etx* coding sequence (CDS). Among the 6 SNPs, 4 are located in the signal peptide, which is removed upon export of the protoxin (proETX), and one is located to the C-terminal, which is removed upon activation via proteolytic cleavage. (**A**) Single nucleotide variants (SNV/SNP) in the CDS of the *etx* gene. Shaded line indicates the least conserved mutation among 6 SNPs. (**B**) The scheme shows location of mutations and key structural features of ETX, with numbers above indicating nucleotide position (numbers in black refer to CDS) and numbers below indicating to amino acid (aa) residues (numbers in orange). Of note, five SNPs, including four mutations in the signal peptide and one mutation in the C-terminal, are removed from the activated toxin by trypsin or intestinal carboxypeptidases. (**C**) Alignment of *etx* reference sequences with complete CDS shows the region (nt 745-777) in which site 762 is centered. Of note, the only *etx* sequence isolated from a human fecal source carries a minor variant at 762 (G). Names of the reference sequences are organized as such: gene name, GeneBank#/NCBI Reference#, toxinotype of *C. perfringens* form which *etx* was sequenced, and followed by animal from which the organism was isolated. (**D)** Alignment of sequences of PCR products from HITMS samples, with the nucleotide corresponding to 762 at the center. PCR products from two reference strains were also included in the sequencing and alignment analysis. Of note, two PCR products yielded short sequences, which were sufficient for confirming *etx* homology but failed to cover site 762 and were therefore excluded from the alignment analysis.

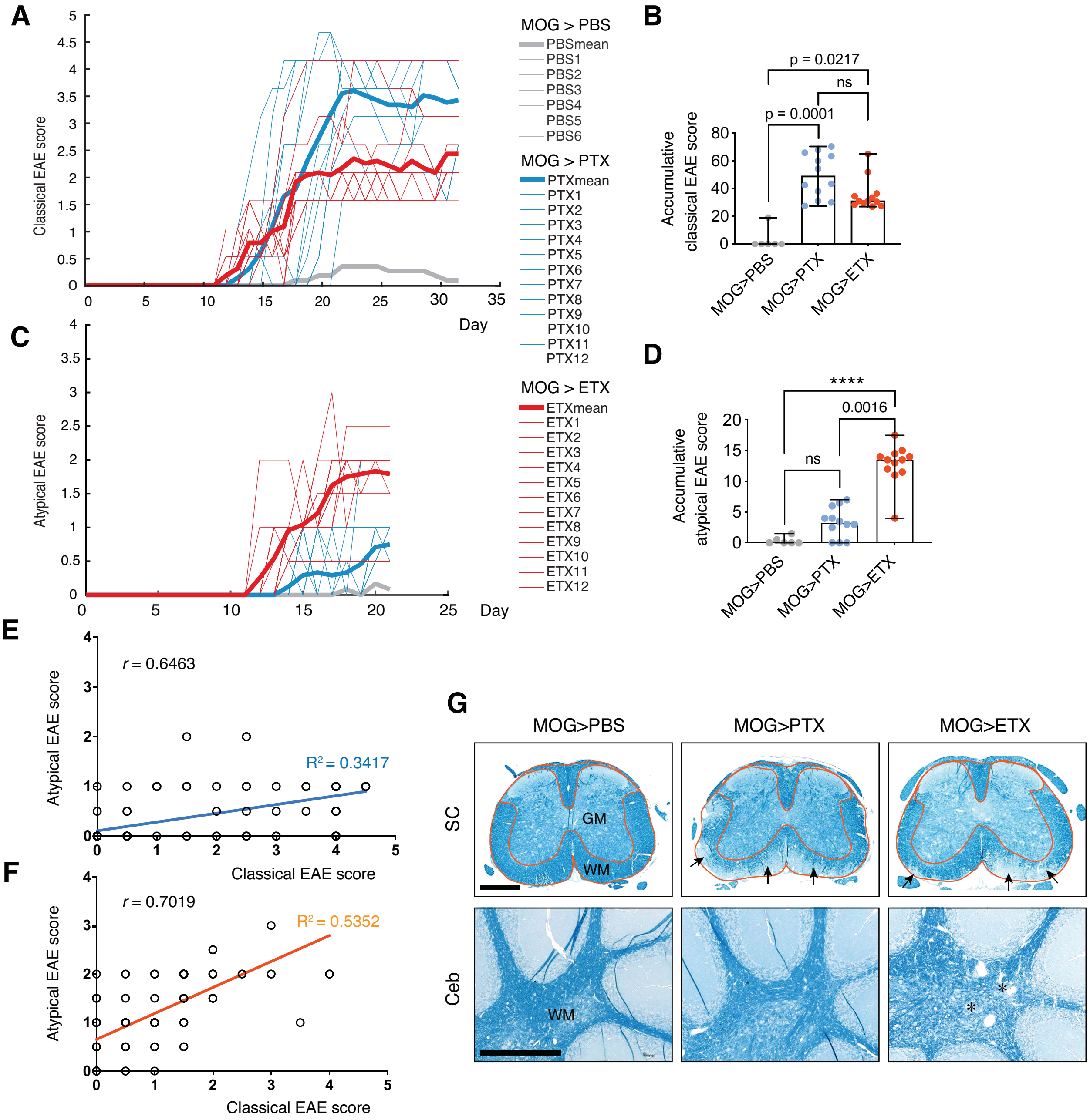

Supplemental Figure 6. **Ascending paralysis and ataxia behaviors are better correlated in ETX-mice than PTX-mice.** Clinical behaviors of classical (**A** and **B**) and atypical (**C** and **D**) EAE. Shown are time-course (A and C) and quantification of accumulative scores (B and D). Of note, atypical EAE (C and D) was not scored after day 21. After day 21, as mice began to experience

more severe and sustained ascending paralysis (A and B), it was not possible to accurately access ataxia, the hallmark of atypical EAE. Thus, the end point of atypical EAE assessment in this experiment was set at day 21. Thick lines in time-course graphs (A and C) indicate mean scores of all mice within the indicated groups (PBSmean; PTXmean; ETXmean) at each daily observing time points. Thin lines depict individual mouse (PBS1-6; PTX1-12; ETX1-12). Note that one mouse from the control group, which received MOG35-55 in CFA but not subsequent injections of either toxin, developed mild EAE. Data in B and D are median ± range; p values determined by Kruscal-Wallis test (non-parametric). ns, not significant; **** $p<0.0001$. n = 6 and 12 mice for the control (MOG>PBS) and experimental groups (MOG>PTX; MOG>ETX), respectively. (**E** and **F)** Analysis on correlation between classical EAE and atypical EAE behaviors in PTX-EAE mice (E, CFA/MOG>PTX) and ETX-EAE mice (F, CFA/MOG>ETX). Data included in E and F were from day 12/13, when at least one type of the clinical phenotypes (classical or/and atypical EAE) initiated, to day 21, when assessment of atypical EAE ended. Spearman correlation analysis; PTX-EAE, $r (108) = 0.6463$, $p < 0.0001$; ETX-EAE, $r (120) = 0.7019$, $p < 0.0001$. Lines in E and F indicate simple linear regression; PTX-EAE (E, blue), $R^2 = 0.3147$; ETX-EAE (F, orange), $R^2 = 0.5352$. Note that many data points (scores) are overlapping in E and F, resulting an appearance of data points (circles) fewer than what were actually included in the analyses (108 and 120 data points for E and F, respectively). (**G)** Representative micrographs of Luxol fast blue (LFB)-stained spinal cord (SC) and cerebellum (Ceb) from all three groups of mice. Orange lines in SC micrographs indicate the edge of spinal cord and the border between gray matter (GM) and white matter (WM). Arrows in SC point to demyelinating area in the ventral funiculus. Asterisks in Ceb indicate demyelination in the white matter. Scale bars, 500 µm. Similar results were achieved in two independently repeated EAE experiments.

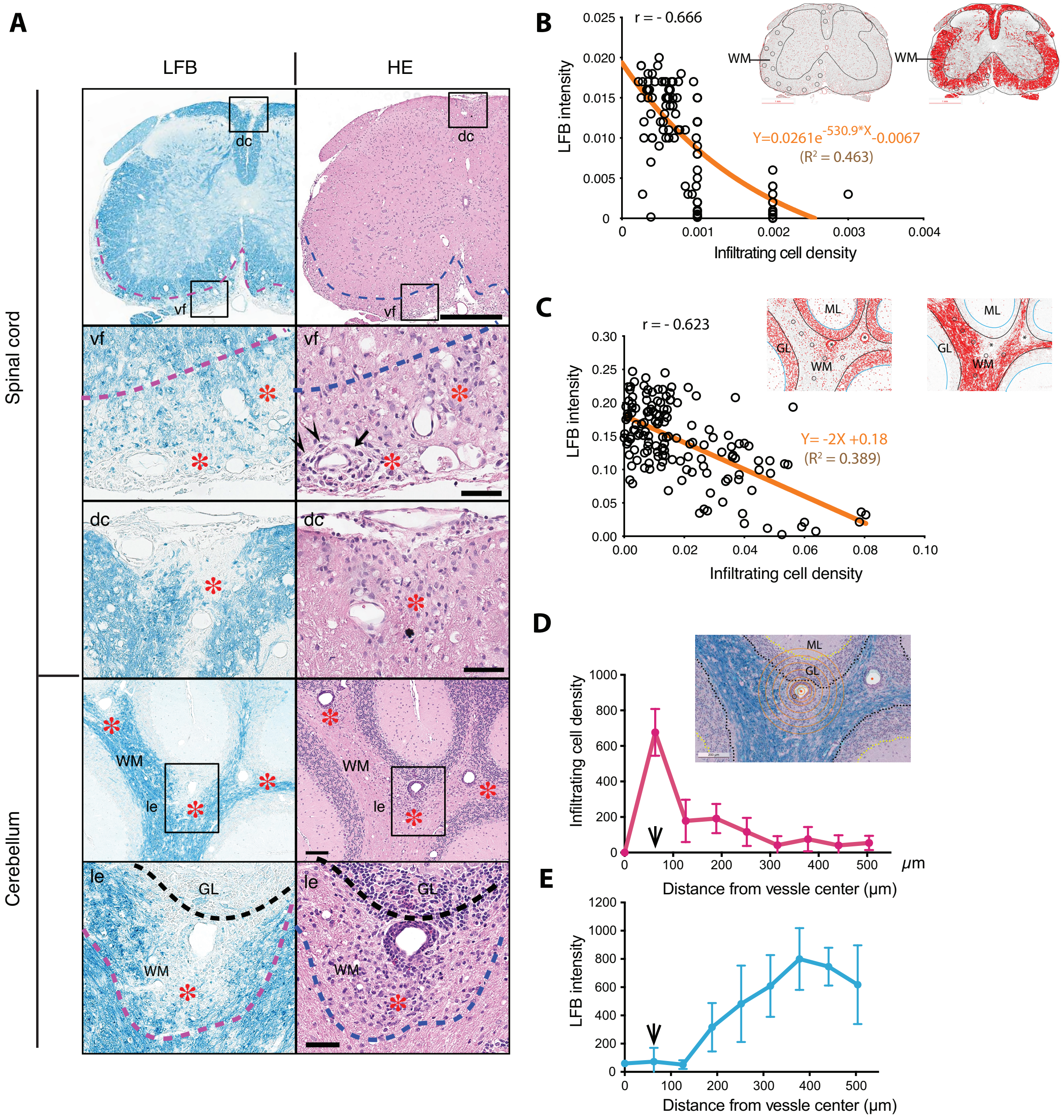

Supplemental Figure 7. **ETX-EAE model is characterized by perivascular demyelination associated with lymphocyte infiltrates.** (**A**) Representative sections from spinal cords and cerebellum from $MOG_{35\text{-}55}$ immunized mice followed by ETX injections were stained with Luxol fast blue (LFB) and consecutive slides were stained with hematoxylin and eosin (HE). Note that

some of the same mice are also shown in Figures 4 and 5. In the spinal cord micrographs, dashed pink dashed lines in LFB stained sections demarcate demyelination lesion in the ventral WM, while dashed blue lines in HE stained sections indicate areas of infiltrating inflammatory cells. High magnification views boxed low-magnification micrographs showing perivascular lesions (red asterisks) in ventral funiculus (vf) and dorsal column (dc). Arrowheads in vf point to the glia limitans or their basement membranes. An arrow points to perivascular space. The cerebellum shows multiple perivascular demyelinating lesions (red asterisks). Boxed regions showing lesions depicted at a higher magnification in below. Black lines in le indicate the border between granular cell layer (GL) and WM. (**B** and **C**) Quantification of LFB intensity and lymphocyte density from the same fields within WM areas in the spinal cord (B) and cerebellum (C) using adjacent sections shows the two variables are negatively correlated by Pearson's correlation analysis. B, r (96) = - 0.666, $p < 0.0001$; C, r (149) = - 0.623, $p < 0.0001$. Best fit curves (orange lines) suggest that LFB signal decays exponentially as the density of lymphocytes increases in the spinal cord WM (B), whereas the two variables are inversely proportional in the cerebellum (C). B, nonlinear regression based on one-phase decay model, $R^2 = 0.463$; C, simple linear regression, $R^2 = 0.389$. Inserts in B and C are binary images generated by thresholding; left, spinal cord section stained with HE; right, an adjacent section stained with LFB. Counts of hematoxylin-stained nuclei (x axis) and LFB staining intensity (y axis) from an arbitrarily chosen region (circle) on adjacent sections were measured. n = 96 and 149 regions in B and C, respectively. (**D** and **E)** Quantification of lymphocyte density (D) and LFB intensity (E) as a function of radial distance from the center of perivascular lesions in WM tracts in the cerebellum confirms an inverse relationship between myelin content and the number of infiltrating lymphocytes. Arrows point to a distance where the perivascular cuff is located. Insert showing a superimposed image of LFB and HE stains illustrates the scheme for

quantification. ML, molecular layer, GL, granular cell layer. Note that ML and GL are excluded from the quantification. Scale bars in A, 1 mm (spinal cord, low magnification), 100 μm (vf, dc), 200 μm (cerebellum, low magnification), 100 μm (le); scale bar in D (insert), 200 μm.

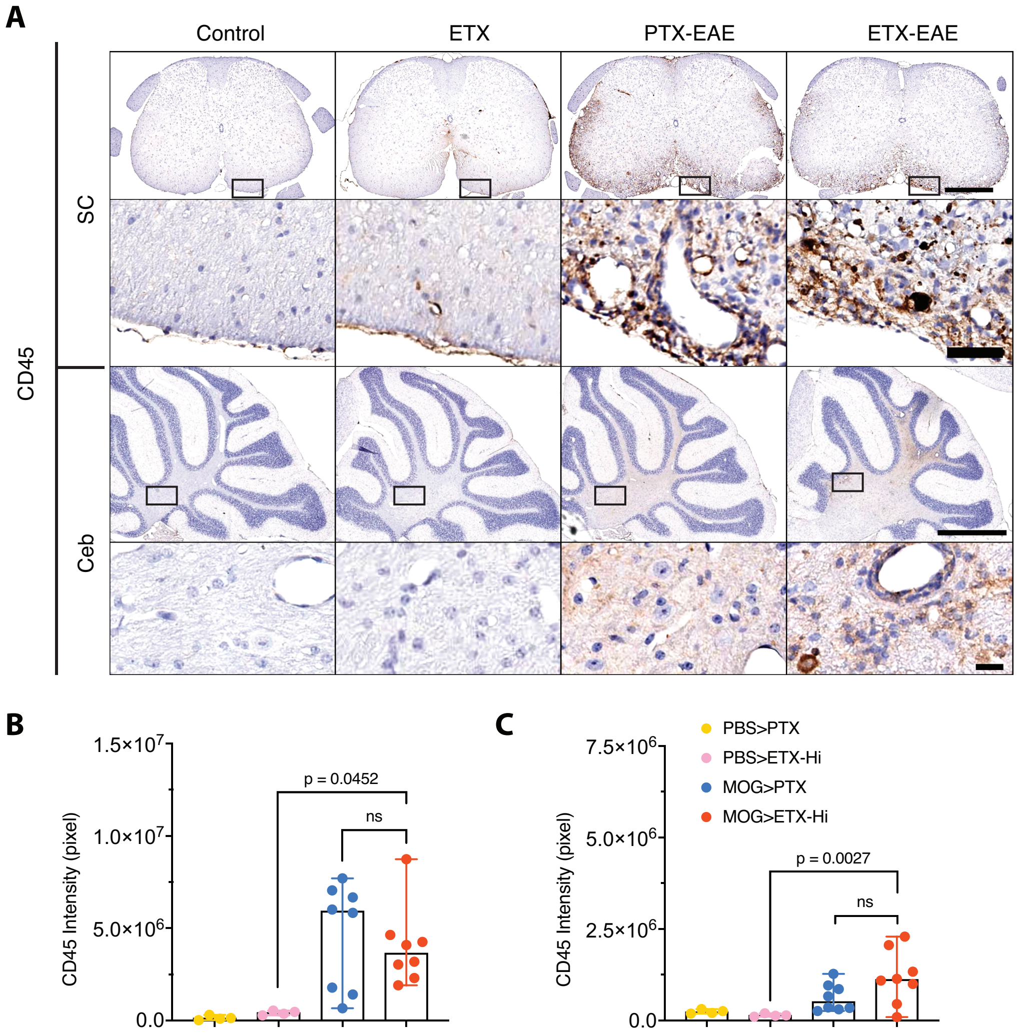

Supplemental Figure 8. **A similar number of CD45-positive cells infiltrate lesions in the spinal cord and cerebellum from ETX-EAE and PTX-EAE mice.** (**A**) Representative sections from mice sacrificed at day 30 post-immunization with CFA/$MOG_{35\text{-}55}$ and followed by either ETX or PTX were immunostained with anti-CD45 antibody. Control mice were immunized with CFA/PBS and followed by either ETX or PTX. A rectangle-framed region in each condition in panel A is shown at a higher magnification beneath the corresponding section. SC, spinal cord; Ceb, cerebellum. Scale bars represent 500 and 50 µm for the spinal cord and 1 mm and 20 µm for the cerebellum at low-magnification and high magnification, respectively. (**B** and **C**) Statistical

analysis of staining intensity for CD45 in the spinal cord (B) and cerebellum (C). Data represent median ± range; p values determined by Kruscal-Wallis test (non-parametric). ns, not significant; n = 4 and 8 mice for controls (Control: PBS>PTX; ETX: PBS>ETX-Hi) and EAE groups (PTX-EAE: MOG>PTX; ETX-EAE: MOG/ETX-Hi), respectively.

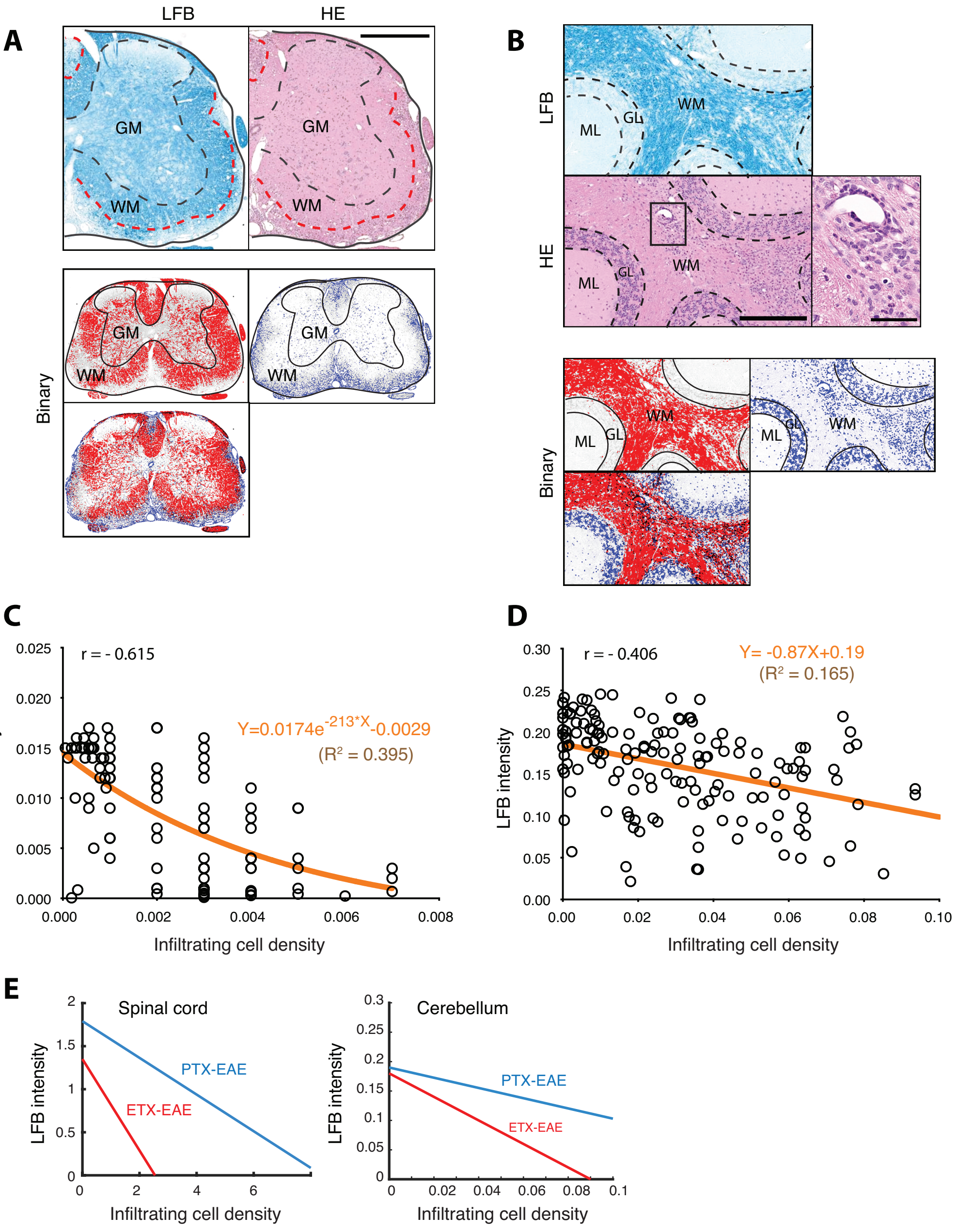

Supplemental Figure 9. Correlation analysis of demyelination and infiltrating lymphocytes in the PTX-EAE model. (**A** and **B**) Representative LFB and HE stained micrographs and corresponding binary images of spinal cord (A) and cerebellum (B) from $MOG_{35\text{-}55}$ immunized mice followed with PTX injections. Note that a perivascular region in the cerebellum (framed with a rectangle in B) is shown at a higher magnification to illustrate morphologies of infiltrating cells.

Binary images were generated by thresholding. For binary images, red corresponds to LFB stain (myelin); blue corresponds to inflammatory infiltrate in hematoxylin stain. GL, granular cell layer; GM, gray matter; WM, white matter. Scale bars, 1 mm (A), 500 µm (B, lower magnification), 100 µm (B, higher magnification). (**C** and **D**) Representative quantitative analyses of demyelination and lymphocyte infiltrates in the spinal cord (C) and cerebellum (D) from MOG_{35-55} immunized mice followed with PTX injections. Pearson's correlation analysis reveals a negative correlation between demyelination and lymphocyte infiltrates in both spinal cord (A) and cerebellum (B). C, r (105) = - 0.615, p < .0001; D, r (154) = - 0.406, p < .0001. Best fit curves (orange lines) suggest a much weaker correlation of the two variables in the cerebellum (D) compared to the spinal cord (C). C, nonlinear regression based on one-phase decay model, R^2 = 0.395; D, simple linear regression, R^2 = 0.165. Counts of hematoxylin-stained nuclei (x axis) and LFB staining intensity (y axis) from an arbitrarily chosen region on adjacent sections were measured. n = 105 and 154 regions in C and D, respectively. (**E**) Slope analyses of myelin intensity versus infiltrating cell density in the spinal cord and cerebellum from ETX-EAE and PTX-EAE mice. Results indicated ETX-EAE model shows a stronger correlation between demyelination and lymphocyte infiltration compared to the PTX-EAE model.

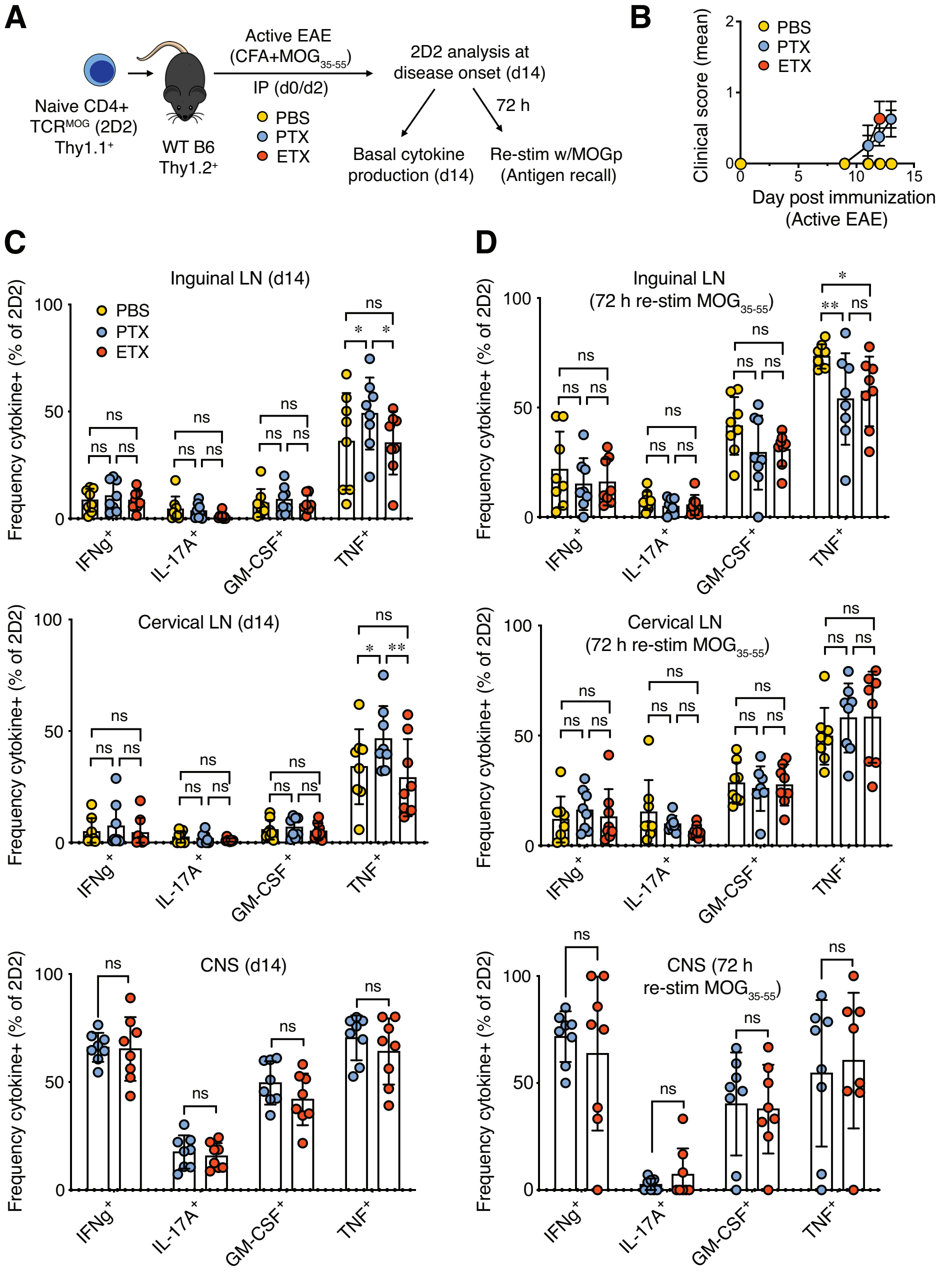

Supplemental Figure 10. **ETX does not alter MOG$_{35-55}$-specific CD4 T cell cytokine responses during active EAE immunization. (A)** Schematic of experiment and analysis of MOG$_{35-55}$-specific T cell responses. Naïve MOG$_{35-55}$-specific CD4 T cells (2D2) were pre-transferred into recipient wild-type B6 mice 24 hours prior to induction of active EAE by subcutaneous

immunization with CFA and $MOG_{35\text{-}55}$, followed by intraperitoneal injection with: PBS, 10 ug/kg PTX, or 500 ng/kg ETX 48 hours after immunization. (**B**) Clinical scores were tracked until onset of clinical disease in PTX and ETX treated mice. (**C-D**) At day 14 post-immunization, single cell suspensions were isolated from indicated tissues, including languid lymph nodes (LN), cervical LN, or CNS (pooled brain and cervical spinal cord), and analyzed by flow cytometry for frequency of cytokine producing 2D2 T cells ($Thy1.1^{+}$) at time of takedown (C) or separately re-stimulated *ex vivo* with $MOGp_{35\text{-}55}$ for 72 hours to determine antigen-recall induced cytokine production (D). Data in B-D are pooled from two independent experiments with similar results (n = 10 mice/group pooled from two independent experiments). Results are shown as mean ± SD. p-values calculated by two-way analysis of variance (ANOVA) with Sidak's multiple comparisons test. Resultant reported as ns (non-significant), $^{*}p < 0.05$, or $^{**}p < 0.01$.

References

1. Carman RJ, Sayeed S, Li J, Genheimer CW, Hiltonsmith MF, Wilkins TD, et al. Clostridium perfringens toxin genotypes in the feces of healthy North Americans. *Anaerobe.* 2008;14(2):102-8.
2. Nagpal R, Ogata K, Tsuji H, Matsuda K, Takahashi T, Nomoto K, et al. Sensitive quantification of Clostridium perfringens in human feces by quantitative real-time PCR targeting alpha-toxin and enterotoxin genes. *BMC Microbiol.* 2015;15:219.
