# Supplementary material for: Epsilon toxin–producing Clostridium perfringens colonize the multiple sclerosis gut microbiome overcoming CNS immune privilege
Source: J Clin Invest. 2023 May 1;133(9):e163239. doi: 10.1172/JCI163239 (PMC10145940; doi:10.1172/JCI163239)
Supplement: Supplemental Methods [file jci-133-163239-s174.pdf]

Supplemental Methods for

Epsilon toxin-Producing *Clostridium perfringens* Colonize the MS Gut and Epsilon Toxin Overcomes Immune Privilege

Yinghua Ma[1]*, David Sannino[1]*, Jennifer R. Linden[1]*, Sylvia Haigh[1], Baohua Zhao[1], John B. Grigg[2,3,4,5], Paul Zumbo[6,7], Friederike Dündar[6,7], Daniel Butler[7], Caterina P. Profaci[8], Kiel Telesford[1], Paige Winokur[9], Kareem Rashid Rumah[10], Susan Gauthier[11], Vincent A. Fischetti[10], Bruce A. McClane[12], Francisco A. Uzal[13], Lily Zexter[11], Michael Mazzucco[1], Richard Rudick[14], David Danko[7], Evan Balmuth[1], Nancy Nealon[11], Jai Perumal[11], Ulrike Kaunzner[11], Ilana L. Brito[15], Zhengming Chen[16], Jenny Z. Xiang[17], Doron Betel[6,7], Richard Daneman[8], Gregory F. Sonnenberg[2,3,4,5], Christopher Mason[7]□, and Timothy Vartanian[1,5,11,18]✉

REAGENTS AND RESOURCES

Reagent	*Source*	*Identifier/Catalogue number*
**Chemicals, peptides, and recombinant proteins**		
Epsilon protoxin, from *Clostridium perfringens*, Strain 34 (Type B)	BEI Resources*	NR-856
Immobilized TPCK Trypsin	Thermo Scientific	20230
Lyophilized rat/mouse MOG 35-55 peptide (Ac/Amide)	AnaSpec	AS-60130-5
Bordetella pertussis toxin, lyophilized	List Biological	181
Heat-killed *M. tuberculosis* H37 Ra (TB)	BD Difco	BD231141
M. butyricum-containing complete Freund's adjuvant	BD Difco	263810
Paraformaldehyde	Thermo Scientific	416785000
EM fixative	NYU EM Core	
Nycodenz	AlereTechnologies	1002424
Phenol:Chloroform:Isoamyl alcohol	Thermo Fisher	BP1752L
Proteinase K	Sigma	P2308
Lysozyme	Thermo Fisher	89833
RNase A	Thermo Fisher	EN0531
Rabbit anti-CD4 monoclonal antibody [EPR19514]	Abcam	Ab183685
Rat anti-CD45 monoclonal antibody [13/2.3]	Abcam	Ab10588
Rabbit anti-CD68 polyclonal antibody	Abcam	Ab125212
Rabbit anti-p-NFkB p65 (Ser 276) polyclonal antibody	GeneTex	GTX55113
Anti-ETX antibody for WB	Vartanian Lab (16)	JL008
Anti-ETX neutralizing antibody	Vartanian Lab (16)	JL004
**Experimental models: organisms/strains**		
Mouse: C57BL/6J	The Jackson Laboratory	RRID: IMSR_JAX:000664

Clostridium perfringens type A	American Type Culture collection MS patient isolate	ATCC13124
Clostridium perfringens type B	American Type Culture collection	ATCC3626
Clostridium perfringens type B	The Uzal Laboratory	NCTC3110
Clostridium perfringens type D	Vartanian Laboratory MS patient isolate	SHDS0050
Clostridium perfringens type D	The Uzal Laboratory	NCTC8346
Clostridium perfringens type D	The Uzal Laboratory	CN3842
Clostridium perfringens type D	The Uzal Laboratory	FU17
Clostridium perfringens type F	American Type Culture collection MS patient isolate	ATCC12915
CHO cells expressing GFP protein	Vartanian Lab (18)	GFP-CHO
CHO cells expressing human MAL-GFP fusion protein	Vartanian Lab (18)	hMAL-CHO

Software and algorithms

ImageJ version 1.53	NIH	https://imagej-nih-gov.ezproxy.med.cornell.edu/ij/index.html
Adobe Photoshop 2020	Adobe	
Adobe Illustrator 2020	Adobe	
Excel	Microsoft	
GraphPad Prism software version 8.0	GraphPad Software	https://www.graphpad.com/scientific-software/prism/
Flye Assembler 2.8.1	University California San Diego	https://github.com/fenderglass/Flye
	St. Petersburg State University	
SPAdes Assembler 3.13.0	OpenGene	https://cab.spbu.ru/software/spades/
Fastp 0.20.0	Wellcome Sanger Institute	https://github.com/OpenGene/fastp
Circlator 3.0	Broad Institute	https://github.com/sanger-pathogens/circlator
Pilon 1.23	Oxford Nanopore	https://github.com/broadinstitute/pilon
Medaka 1.0.3	Technologies	https://github.com/nanoporetech/medaka
	Heng Li	
Burrows-Wheeler Aligner 0.7.17	The Darling Laboratory	https://github.com/lh3/bwa
Mauve	GView Server	http://darlinglab.org/mauve/mauve.html

GView BLAST Atlas	https://server.gview.ca/

* This reagent was obtained through BEI Resources, NIAID, NIH: Epsilon protoxin, from *Clostridium perfringens*, Strain 34 (Type B), NR-856.

Further information and requests for resources and reagents should be directed to and will be fulfilled by the Lead contact, Timothy Vartanian (tiv2002@med.cornell.edu).

METHODS

Recruitment of participants for Harboring the Initial Trigger of Multiple Sclerosis (HITMS) study and IRB (# 1003010940)

Patients were prospectively screened for eligibility for the HITMS study by Weill Cornell MS Center Clinical Coordinators and eligible participants were provided with the study synopsis and the informed consent form to review. HC were recruited by advertising through flyers, website announcements, and recruiting friends of patients (genetically unrelated). Participants wishing to join the study completed and signed the informed consent in the presence of IRB approved personnel within the MS Center. Enrolled participants were assigned a study number, provided a fecal collection kit consisting of two biohazard bags, 6 x 50 ml sterile conical polypropylene tubes, one freezer box, a blue pad, a fecal collection toilet hat, sterile tongue depressors, and instructions on self-collection of fecal samples.

Criteria for inclusion/exclusion of HITMS study participants and sample size

Inclusion criteria: 1. Participants with clinically definite multiple sclerosis (MS) (1) – male and female participants 18 years of age or older who have been accurately diagnosed with MS based on revised McDonald criteria (1). These subjects must have the ability to provide consent and be willing to participate in the study. 2. Healthy controls were enrolled for comparison.

Exclusion criteria: Any participant who met the criteria below was excluded from participating in this study: 1. Inability to provide informed consent (2). 2. Any form of dementia or cognitive impairment (2). 3. Current or chronic use of anticoagulants. 4. Pregnancy. 3. Body mass index greater than or equal to 39 or less than or equal to 17.5. 5. Use of the following medications within the last 6 months: a) Systemic antibiotics (intravenous, intramuscular, or oral) for greater than 3

days, b) Amylase inhibitors, c) Commercial probiotics consumed at cfu's > 10^8 organisms per day). 6. Chronic immunodeficiency, renal, metabolic, pancreatic, hepatic, gastrointestinal (Crohn's Disease, Ulcerative colitis), pulmonary, or cardiovascular disease requiring ongoing treatment. 7. Hematologic disease, derangements, blood dyscrasias, unrelated to standard of care MS treatments. 8. Major dietary changes (e.g., omnivore to vegan, vegan to omnivore) in the 3 months prior to fecal sample collection. 7. Chronic alcohol consumption defined as more than 5 oz (or 5 drinks) or ethanol per day. 8. Any history of fecal microbiota transfer. Many of the above criteria were based on the NIH Human Microbiome Project Core Microbiome Sampling Protocol A: https://www.ncbi.nlm.nih.gov/projects/gap/cgi-bin/study.cgi?study_id=phs000228.v3.p1

Sample size: We based the power calculation on a dichotomous result of being *etx*- positive or negative. We estimated the incidence of being *etx*-positive to be 10% in the HC group and 40% in the MS group. We set Alpha at 0.05, Beta at 0.2, and a Power of 0.8. This resulted in a calculated sample size of 62 participants with 1:1 enrolment of 31 HC and 31 MS participants.

Fecal microbiota separation and DNA extraction

Stool samples were collected from 31 MS patients and 31 healthy donors and stored in a locked -80°C freezer. Frozen samples were thawed in a Whitley A35 HEPA workstation set at 37°C with 40% humidity for an hour. A part of each stool sample was subjected to Nycodenz density gradient centrifugation for separating the microbiota from other fecal materials (3), and each fecal sample was sampled at minimum 3 times. Two grams of feces were aseptically transferred with a sterile tongue depressor to 18 mL of 0.9% NaCl prepared in ultrapure water, containing 16, 2 mm sterile metal beads, and homogenized by vortexing for 2 minutes. 10.5 mL of homogenized feces were added onto the top of 3.5 mL of 80% (w/v) Nycodenz in ultrapure water, and centrifuged at 10,000 g for 1 hour at 4 °C. The layer corresponding to microbiota was collected, washed twice with 1 mL PBS, and resuspended in 1 mL PBS. Bacteria were digested with lysozyme, RNase A, and proteinase K, and lysed with sodium lauryl sarcosinate (sarkosyl). From lysates DNA was isolated with phenol:chloroform:isoamyl alcohol (25:24:1) and DNA was precipitated with 100% ethanol. The DNA pellet was washed with 70% ethanol and resuspended and in sterile ultrapure water for subsequent standard PCR and quantitative PCR analyses.

C. perfringens reference strains, including ATCC 3626, ATCC 13124, and ATCC12915, were grown on Rapid Perfringens Medium (RPM) at 37 °C overnight. Bacteria were harvested by centrifugation at 4000 rpm for 10 min at 4 °C and followed by total DNA extraction as descried above. DNA from reference strains were included in both standard PCR and quantitative PCR analyses as controls or calibrators.

Detection of the *etx* gene and toxinotyping of *C. perfringens* communities

For the detection of *etx* and other genes indicated in the study, simplex and multiplex PCRs were performed with DNAs extracted from fecal microbiota using Platinum II Hot Start OCR Master Mix kit (Thermo Fisher # 14000014) following the manufacturer's instructions and using the PCR primers and parameters that follow.

Primers and parameters on PCR analysis

For the detection of *etx* and other genes indicated in the study, simplex PCR includes the following primers: *etx* (3' terminal), forward: 5'-ACTGCAACTACTACTCATACTGTG-3', reverse: 5'-CTGGTGCCTTAATAGAAAGACTCC-3'; *etx* (5' terminal), forward: 5'-GCATCAGCGGTGATATCCATC-3', reverse: 5'-TCTCTCCCCATTCACTTCCAC-3'; *cpa/plc*, forward: 5'-GTTGATAGCGCAGGACATGTTAAG-3', reverse: 5'-CATGTAGTCATCTGTTCCAGCATC-3'; Universal 16S rRNA, forward (8F): 5'-AGAGTTTGATCCTGGCTCAG-3', reverse (1492R): 5'-GGTTACCTTGTTACGACTT-3'; *C. perfringens*-specific 16S rRNA, forward: 5'-AGATGGCATCATCATTCAAC-3', reverse: 5'-GCAAGGGATGTCAAGTGT-3'.

Toxinotyping of *c. perfringens* in the fecal microbiota was performed using a modified multiplex protocol based on a recent report (4). Primers included in the multiplex PCR include the following: *etx* (5'-terminal): *etx* (5' terminal, 697 bp), forward: 5'-GCATCAGCGGTGATATCCATC-3', reverse: 5'-TCTCTCCCCATTCACTTCCAC-3'; *cpa/plc* (402 bp), forward: 5'-GTTGATAGCGCAGGACATGTTAAG-3', reverse: 5'-CATGTAGTCATCTGTTCCAGCATC-3'; *cpb* (236 bp), forward: 5'-ACTATACAGACAGATCATTCAACC-3', reverse: 5'-TTAGGAGCAGTTAGAACTACAGAC-3'; *itx* (317 bp): forward, 5'-

GCGATGAAAAGCCTACACCACTAC-3’, reverse, 5’-GGTATATCCTCCACGCATATAGTC-3’; *cpe* (506 bp), forward: 5’-GGGGAACCCTCAGTAGTTTCA-3’, reverse: 5’-ACCAGCTGGATTTGAGTTTAATG-3’. For both simplex and multiplex PCRs, *C. perfringens* reference strains, including ATCC 3626 for type B and FD203 for type D, were used as the positive control for *etx*, whereas reference strains, including ATCC 13124 for type A and ATCC12915 for type F, served as the negative controls for *etx.*

The amplification program used for all assays started with 94 °C for 5 min and followed by 35 cycles of 45 sec at 94 °C, 1 min at 50-58 °C (for simplex PCRs: 57 °C for 5’-*etx*, 53 °C for 3’-*etx*, 58 °C for *cpa*, 50 °C for universal 16S rRNA, 55 °C for *C. perfringens*-specific 16S rRNA; for multiplex PCR: 55°C), 1 min at 68 °C, and a final extension step of 10 min at 68 °C. . The PCR products were electrophoresed on 1.2% agarose gel and visualized by an Azure c200 Gel Imaging System.

Quantitative analysis of *etx* gene abundance and proportion of *etx*-harboring *C. perfringens* in the fecal microbiota

Quantitative PCR (qPCR) was performed on an Applied Biosystems QuantStudio 6 Flex Real-Time PCR System (Thermo Fisher) using PerfecTa Multiplex qPCR SuperMix kit (Quanta Bio # 95108-200) following the manufacture’s instruction. Custom-designed target-specific TaqMan probes labeled with FAM/VIC and quenched with TAMRA/MGBNFG were utilized. In each qPCR system, amplicons were designed to be of similar sizes, and primers for the target sequences having similar melting temperatures were elected to achieve comparable amplification efficiency. Universal 16S rRNA served as a reference gene in most cases. All measurements were performed in triplicate. The specificity of each qPCR system was tested and verified by using *etx*-harboring or non-*etx C. perfringens* reference strains as positive and negative controls.

Relative abundance of *etx*, *cpa*, and *C. perfringens*-specific 16S rRNA genes was determined by setting universal 16S rRNA as a reference gene using cycle of threshold (Ct) and a $2^{-\Delta Ct}$ algorithm (5). Further, percentage of *C. perfringens* among fecal microbiota was computed using lab reference strain culture (100%) as calibrators based on a $2^{-\Delta\Delta Ct}$ algorithm (5). Similarly, ratios of *etx*-harboring strains over non-*etx* strains were assessed using *etx/cpa* from a reference *etx*-harboring strain (type D). PCR primers and parameters for qPCR analysis follow.

Primers and parameters on qPCR analysis

qPCR for *etx* abundance include the following primers and fluorogenic probes: *etx*, forward: 5'-CATACTGTGGGAACTTCGATACA-3', reverse: 5'-TCTTGTGAAGGGACATTATGAGTAA-3', probe: 6-FAM-AGCAACTGCTAAGTTTACTGTTCCT-TAMRA; Universal 16S rRNA, forward, 5'-GCGAGACTGCCGGTAATAAA -3', reverse, 5'- TCGTTGTACCAGCCATTGTAG -3', probe: VIC - CCCTTATGACCTGGGCTACACACG – MGBNFQ. A PCR cycling protocol consisting of 45 sec at 94 °C, 1 min at 62 °C, and 30 sec at 68 °C for 40 cycles.

qPCR for *cpa* abundance include the following primers and fluorogenic probes: *cpa*, forward: 5'- CTTGGAGAGGCTATGCACTATTT -3', reverse: 5'- TTGCAACCTGCTGTGTTTATTT -3', probe: 6-FAM-TTACTGCCGTTGATAGCGCAGGAC-TAMRA; Universal 16S rRNA, forward, 5'- GCGAGACTGCCGGTAATAAA -3', reverse, 5'-TCGTTGTACCAGCCATTGTAG -3', probe: VIC - CCCTTATGACCTGGGCTACACACG – MGBNFQ. A PCR cycling protocol consisting of 45 sec at 94 °C, 1 min at 62°C, and 30 sec at 68 °C for 40 cycles.

qPCR for *C. perfringens* abundance include the following primers and fluorogenic probes: *C. perfringens*-specific 16S rRNA, forward: 5'-AGATGGCATCATCATTCAAC-3', reverse: 5'-GCAAGGGATGTCAAGTGT-3', probe: 6-FAM-AGAGTGCAGGAGAGGAGAGTGGAA - TAMRA; Universal 16S rRNA, forward, 5'- GCGAGACTGCCGGTAATAAA -3', reverse, 5'-TCGTTGTACCAGCCATTGTAG -3', probe: VIC - CCCTTATGACCTGGGCTACACACG – MGBNFQ. A PCR cycling protocol consisting of 45 sec at 94 °C, 1 min at 58 °C, and 1 min 15 sec at 68 °C for 40 cycles.

qPCR for *etx/cpa* ratio include the following primers and fluorogenic probes: *etx*, forward: 5'-CATACTGTGGGAACTTCGATACA-3', reverse: 5'-TCTTGTGAAGGGACATTATGAGTAA-3', probe: 6-FAM-AGCAACTGCTAAGTTTACTGTTCCT-TAMRA; *cpa*, forward: 5'- GCATGAGTCATAGTTGGGATGA -3', reverse: 5'-CTGATGGATCATTACCCTCTGATAC -3', probe: VIC-

TGGGACTATGCAGCAAAGGTAACTTTAGC -MGBNFQ. A PCR cycling protocol consisting of 45 sec at 94 °C, 1 min at 62 °C, and 30 sec at 68 °C for 40 cycles.

Bacterial strains and culture

Multiple *C. perfringens* strains were utilized in this study. The type A (ATCC13124), B (ATCC3626), and F (ATCC12915) strains were purchased from ATCC. Four other collection strains were provided by Francisco Uzal: 3 type D strains, CN3842, NCTC8346, and FU17, and 1 type B strain NCTC3110. The type D strain FU17 was isolated from the gut of a goat with clinical enterotoxaemia, including brain perivascular edema (6). All strains, including the type D strain SHDS0050 isolated in this study, were grown, and maintained at 37°C with 40% humidity in a Whitley A35 HEPA anaerobic workstation in rapid perfringens media (RPM) (3% fluid thioglycolate medium, 6% gelatin, 0.5% peptone, 0.5% dextrose, 0.5% potassium phosphate dibasic. 0.3% yeast extract, 0.15% sodium chloride 0.05% ferrous sulfate, 440mg/mL D-cycloserine) (7).

Genome sequencing preparation and analysis

To understand the *etx* plasmid architecture of the SHDS0050 strain and compare it to other *etx*-producing strains, pure cultures of *C. perfringens* strains ATCC 3626, CN 3842, FU17, NCTC 3310, NCTC 8346, and SHDS0050, were grown overnight in RPM, and total genomic DNA was isolated from each strain. DNA library preparations were made for both Illumina (short reads) and Oxford Nanopore sequencing (long reads) with 50X coverage for each. Illumina libraries were generated using the Nextera Flex Protocol (now renamed Illumina DNA Prep).

250 ng of genomic DNA for each sample was diluted into 10 µL and taken into library prep. DNA was fragmented, cleaned, and amplified using IDT indexes for multiplexing. Samples were run on a NovaSeq S4 Flow Cell at PE150 and reads were demultiplexed using Illumina BaseSpace software. Nanopore libraries were generated using the LSK-109 ligation sequencing kit from Oxford Nanopore and run on the PromethION sequencing device. Briefly, 1ug of DNA was diluted into 48 µL and taken into library prep using the LSK-109 kit from Oxford Nanopore. Adapters were ligated to the DNA, followed by motor proteins. This library was loaded onto a PromethION Flow Cell PRO-002 and allowed to run for 64 hours. Reads were demultiplexed

using Guppy software from Oxford Nanopore built in to the PromethION device. Nanopore reads were assembled using the Flye 2.8 assembler (8), with 10 iterations. Medaka 1.0.3 (Oxford Nanopore Technologies Ltd.) was used to polish the Flye assemblies with the Nanopore reads. To further clean the assemblies, Illumina reads were trimmed and quality controlled via Fastp 0.20.0 (9) and were mapped onto the Medaka polished genome using the Burrows-Wheeler Aligner (BWA) 0.7.17 (10, 11). These alignments were used to further polish the genome with Pilon 1.23 (12). Further, both Illumina and Nanopore reads were assembled in a hybrid assembly with SPAdes 3.13 (13, 14). Chromosomes were circularized using Circlator 3.0(15) or by aligning the SPAdes assemblies to the more contiguous Flye assemblies. To ensure that plasmids were circular, plasmid sequences from the polished Flye assemblies were aligned to the SPAdes assemblies using Mauve(16), and the SPAdes assembly was used to fill in the gaps to circularize the plasmids. Chromosome assemblies were compared using the BLAST Atlas function of GView(17).

Bacterial culture conditions for pETX production

Frozen cultures stored at -80°C in 50% RPM / 50% glycerol stocks were streaked onto BBL™ Schaedler Agar with Vitamin K1 and 5% Sheep Blood (BD) and placed in BD GasPak EZ anaerobe pouch system grown at 37°C for at least 48 hours. Large inoculums were used to start 13 mL RPM cultures incubated at 37°C for six hours under anerobic conditions. 3 mL of the 6-hour RPM cultures were used to inoculate 10 mL of TGY broth (3% tryptic soy broth, 1% yeast extract, 0.1% sodium thioglycolate). TGY cultures were incubated overnight at 37°C. To harvest conditioned media, overnight TGY cultures were centrifuged at 12,000rcf for ten minutes and supernatant carefully collected without disturbing bacterial pellets. Harvested media was stored at -20°C until use. Sterile broth was used as negative controls. Note, when this protocol was used, a direct inoculation into TGY broth did not result in growth.

Western blot analysis of proETX production

A total of 10 µL of conditioned TGY broth were loaded onto gels. 10 µL of sterile TGY broth was used as a negative control. 10 ng of pETX in 10 µL of PBS or TGY broth were used as positive controls. All samples were prepared in 2X Laemmli Sample Buffer (Bio-Rad) containing 5% 2-Mercaptoethanol (Bio-Rad) and heated at 95°C for 5 min before loading onto 4–20% Mini-PROTEAN TGX Stain-Free gels (Bio-Rad). 5 µL per lane of WesternSure Pre-stained

Chemiluminescent protein ladder (Licor) were used as molecular weight standards. Gels were run in Tris/Glycine SDS Buffer (Bio-Rad) at 200 V for 30 min. Semi-dry transfers were performed in transfer Tris/Glycine Buffer (Bio-Rad) using the Trans-Blot SD Semi-Dry Electrophoretic Transfer Cell system (Bio-Rad) at 15 V for 15 min. Blots were blocked in 5% Blotting-Grade Blocker nonfat milk (Bio-Rad) in Tris Buffered Saline with Tween 20 (TBS-T, Cell Signaling Technology) for 30 minutes at room temperature. Blots were then incubated with anti-ETX antibody JL008 (18) at 0.2 µg/mL in blocking solution overnight at 4°C. Blots were washed with TBS-T at room temperature and incubated with secondary antibody peroxidase-conjugated Affinipure Goat Anti-Rabbit IgG H + L (Jackson ImmunoResearch) at 0.024 µg/mL in blocking solution for 1 hour at RT. Blots were washed again in TBS-T and developed for 5 min at room temperature in SuperSignal West Dura Extended Duration Substrate (ThermoFisher Scientific). The developed blots were visualized on 5x7 CL-XPosure Films (ThermoFisher Scientific) at various exposure times using a Konica Minolta SRX-101A film processor.

ETX activation

Epsilon protoxin (NR-856) obtained from BEI resources was activated with immobilized TPCK Trypsin following the manufacture's protocol (19). Each batch of activated ETX was normalized for activity by the assessment of cytoxicity using a CHO cell line that expresses rMAL (20). Epsilon Protoxin, from *C. perfringens*, Strain 34 (Type B), NR-856 was obtained through BEI Resources, NIAID, NIH.

hMAL-CHO cell sensitivity assay

Harvested TGY broth was incubated with equal amounts 0.25% Trypsin-EDTA solution (Gibco) for 2 hours at 37°C. Sterile TGY broth was used as a control. Trypsin activity was stopped by the addition of FBS to a total FBS percentage of 25% (i.e., 25 µL FBS added to 100 µl of TGY/Trypsin solution). 50 µL of trypsin treated TGY broth were used to treat confluent hMAL-CHO or GFP-CHO cells seeded in 200 µl of CHO cell media (Dulbecco's Modified Eagle's Medium/Ham's F12 medium (Life Technologies) with 10% heat-inactivated fetal bovine serum, Glutamax, and 50 units/ml penicillin and 50 µg/mL streptomycin) in 96 well plates. 50 µL trypsin treated TGY broths were treated with neutralizing anti-ETX antibody JL004 at 50 µg/mL for 20 minutes prior to CHO cell treatment. CHO cells were treated overnight at 37°C. To access cell death, cells were

treated with 50 μg/mL of propidium iodide (PI, Sigma). Live images of randomly chosen fields in each well were acquired under an inverted fluorescence microscope (Nikon, Minato, Tokyo, Japan) equipped with a Charged Coupled Device (CCD) camera (Carl Zeiss, Oberkochen, Germany) imaged with Spot software and were then imported into ImageJ64 in 8-bit gray format. For quantification of PI-positive cells, the images were converted into binary images by applying the same threshold value to all images collected from the same experiment. Analyze Particles function was selected to automatically count the particle numbers Data were exported and analyzed in Excel (Microsoft) and Prism version 9.0.2 (Graphpad).

EAE induction and clinical scoring

8-10-week-old female C57BL/6 mice received subcutaneous (s.c.) injection of 200 μg synthesized mouse/rat MOG_{35-55} (MEVGWYRSPFSRVVHLYRNGK) emulsified in 50 μL of complete Freund adjuvant and supplemented with 200 μg heat-inactivated *M. tuberculosis* H37Ra (TB). On day 0 and 2, 5 μg/kg PTX or ETX at 50 or 500 ng/kg b.w. was administered via intraperitoneal injection. Animals were weighed and scored daily. Assessment of classical EAE was based on a previously published scale (21) and as follows: 0 refers no physical signs of disability; 0.5, loss of tail tone or distal tail limpness; 1, complete tail limpness; 2, both limp tail and weakness/dragging of hind limbs; 3, hind limb paralysis, 4, complete paralysis of hind limbs and partial paralysis of forelimbs; 5, moribund or death. Peak, average, and accumulative scores were calculated to assess EAE severity.

Assessment of atypical EAE was carried out separately from the classical EAE symptom described above, and based on previously published scales (22) with modifications, Specifically: 0, no disease; 1, hunched appearance, stiff tail, slight head tilt; 2, staggered walking, scruffy coat; 3, staggering irregularly and *lurching* from side to side, obvious impaired balance/ambulation, slight axial rotation; 4, Severe axial rotation, spinning, severe body lean, fall; 5, moribund. As diseased mice experienced ascending paralysis, it became increasingly difficult and less certain to evaluate ataxia, the hallmark of atypical EAE. Thus, the end point of atypical EAE assessment was set at a time when classical EAE has reached the peak, which ranged from 17-21 days.

Histological analysis

Mice were anesthetized with ketamine/xylene cocktail and followed by transcardiac perfusion with PBS and 4% PFA. Brains and spinal cords were removed, processed for paraffin-embedding, and sectioned at 5 μm thickness. Sections were stained with hematoxylin and eosin to evaluate the overall morphology and lymphocyte infiltration. The inflammatory parameters were assessed on the following scale: 0, no sign of inflammation; 1, scattered inflammatory cells; 2, some inflammatory cells and karyopyknosis; 3, perivascular inflammatory cell infiltrate; and 4, marked inflammatory cell infiltration into the parenchyma. Consecutive sections were stained with Luxol Fast Blue (LFB) for myelin. The size of demyelinated area and the number of infiltrating inflammatory cells were measured using ImageJ software (National Institutes of Health, USA). A universal threshold was applied to the images across all sections in all conditions. Area of LFB staining intensity was limited to threshold, while the total area of white matter was measured without thresholding. Myelin integrity is defined by the ratio of LFB-stained area within the WM (pixel with thresholding) over the total area of the WM (pixel without thresholding) and expressed as a percentage.

Immunohistochemical analysis

Paraffin-embedded sections from EAE and control mice were submitted to Histowiz (New York NY) for immunohistochemical staining for CD4, CD45, CD68, and phosho-NFκB expression. See reagents in Supplemental Methods. Quantification of staining signal was performed with Image J software (National Institutes of Health, USA) and integrated intensity was used for statistical analysis with Prism 9.

Electron microscopy

Mice were anesthetized with ketamine/xylene cocktail, transcardiacally perfused with 0.1 M PB and EM fixative 4%PFA, 2.5% glutaraldehyde, 0.1M sucrose in 0.1MP. Immediately after perfusion, brains and lumbar spinal cords were removed and cut into 2 mm-thick brain slices and spinal cord segments. The trimmed tissues were Immersed in the above fixative for two days before tissue processing at the Electron Microscopy Core of New York University. Semi-thin sections at 1 μm thickness were cut and stained with toulidine to identify target regions using light microscopy. The target regions were then trimmed and reoriented and embedded in epoxy resin.

Ultrathin (70 nm) cross sections were cut and stained with uranyl acetate and lead citrate, and imaged under transmission electron microscope (JEOL, MA).

Structural analysis on myelin

Electron microscopy analysis was performed to determine changes of myelin sheath in EAE and control mice following a standard protocol. Tissues processing, preparation of semi-thin and ultrathin sections, and imaging were performed at the Electron Microscopy Core of New York University. For quantification, 30-40 electron micrographs from 12 randomly chosen fields from each mouse were imaged at both low (4000 X) and high magnifications (40000 X), among which 12 micrographs of adequate quality were used for analysis with Image J. Parameters used to evaluate demyelination included counts of unmyelinated/demyelinated axons, and morphological abnormalities of myelin sheaths and axons. Unmyelinated/demyelinated axons were defined as an axon of appropriate diameter without at least one complete wrap of an oligodendrocyte process. Demyelination was expressed as an average number of unmyelinated axons per field as well as per area unit (mm^2) measured using Image J. Axon degeneration was assessed based on a previously published classification scheme (23). According to this scheme, degenerated axons are identified as a) myelin profiles that lack an axon (axolysis, either due to vacuolization or to condensation); b) swollen axons lacking organelles and neurofilaments; c) axons that contain swollen mitochondria or mitochondria with disrupted cristae; d) axonal profiles with electron dense cytoplasm likely due to increased cytoskeletal or neurofilament density. Quantification of staining signal was performed with Image J software (National Institutes of Health, USA) and integrated intensity was used for statistical analysis with Prism 9.

Isolation of cells from lymph nodes and CNS of mice and antigen-recall assay

Mice were euthanized and lymph nodes (cervical and inguinal), central nervous system (CNS, brain and spinal cord) were immediately collected by dissection and held on complete RPMI media containing 10% FBS, Penicillin-Streptomycin, L-glu, HEPES, and β-mercaptoethanol. Lymph nodes were dissociated using a syringe plunger passed through cell strainer (70 µm). The CNS was finely minced with a razor blade and digested for 20 minutes at 37°C in incubator shaker with collagenase D (2 mg/ml; Roche Diagnostics) and DNaseI (0.1 mg/ml; Sigma) in HBSS (Sigma Aldrich). Mononuclear cells were further purified by passage through cell strainer (70 µm) and

enriched by 30 over 70% Percoll gradient centrifugation (GE Healthcare). Where indicated, to determine antigen-recall response, bulk cell suspensions were cultured at 37°C for 72 hours with exogenous MOG_{35-55} (50 µg/mL) prior to analysis of cytokine production by flow cytometry.

CNS endothelial isolation, RNA extraction, sequencing, and RNA-sequencing analysis

Mice were treated with PBS, ETX (0.5 µg/kg b.w.), or PTX (5 µg/kg b.w) on two consecutive day. 16 hours after the second dose, CNS endothelial cells were isolated from spinal cords or brains with the cerebellum removed as previously described (24). Brefily, CNS tissue was enzymatically dissociated with a papain solution followed by vigorous trituration and a second dissociation with collangese and dispase solution. Myelin was removed using Miltenyi Biotec Myelin Removal Beads II per the manufacturer's instructions. Isolated cells were stained with anti-CD31 clone 390, anti-CD45 clone 30-F11 (, and CD11b clone M1/70, anti-CD13 clone R3-242, anti-PDGFbeta clone APB%, and DAPI. Viable endothelial cells (DAPI-) positive for CD31 only (CD31+, CD45-, CD11b-, CD13-, and PDGFbeta -) were sorted via FACS using a BD Biosciences FACSAria II Cell Sorter. RNA was extracted from sorted endothelial cells using Qiagen's RNeasy Plus Micro Kit per the manufacturer's instructions. Total RNA integrity was checked using a 2100 Bioanalyzer (Agilent Technologies, Santa Clara, CA). The cDNA synthesis and amplification were performed by SMART-Seq v4 ultra low input RNA kit (Takara Bio USA, Mountain View, CA, USA) starting with less than 1 ng of total RNA from each sample. 150 pg of qualified full-length double-strand cDNA was used and processed for Illumina library construction with the Nextera XT DNA Library Preparation Kits (Illumina, San Diego, CA). Then the normalized cDNA libraries were pooled and sequenced on Illumina NovaSeq6000 sequencer with pair-end 100 cycles. The raw sequencing reads in BCL format were processed through bcl2fastq 2.19 (Illumina) for FASTQ conversion and demultiplexing.

Raw reads were quality checked with FastQC v0.11.7 (http://www.bioinformatics.babraham.ac.uk/projects/fastqc/). Reads were aligned to the mouse reference genome (GRCm38.p6) using STAR v2.7.6a with default parameters (25). Gene abundances were calculated with featureCounts v2.0.1 (26) using composite gene models from Gencode release vM25 (27). Differential expression analysis was performed in R using limma (v3.50.3) (28), after removing lowly expressed genes with the filterByExpr function from edgeR

(v3.36.0) (29). In brief, linear models were fitted with treatment information to create the design matrix, followed by empirical Bayes moderation of t-statistics. Raw P-values were adjusted for multiple testing using the Benjamini & Hochberg method (30), and only genes with an adjusted p < 0.10 were considered differentially expressed. Differentially expressed genes for the ETX vs. PBS contrast were analyzed using Ingenuity Pathway Analysis (IPA, QIAGEN Inc., https://www.qiagenbioinformatics.com/products/ingenuity- pathway-analysis, version 01-21-03). Core Analysis settings included all available data sources from human, mouse, or rat species. Expression heatmaps were generated with pheatmap (R package version 1.0.12. https://CRAN.R-project.org/package=pheatmap) using log2 counts per million (CPM), with the values centered and scaled by row. All scripts and code used for generating the bulk RNA-seq based figures can be found at https://github.com/abcwcm/Vartanian2023.

References:

1. Polman CH, Reingold SC, Banwell B, Clanet M, Cohen JA, Filippi M, et al. Diagnostic criteria for multiple sclerosis: 2010 revisions to the McDonald criteria. *Ann Neurol.* 2011;69(2):292-302.
2. Dunn LB, and Jeste DV. Enhancing informed consent for research and treatment. *Neuropsychopharmacology.* 2001;24(6):595-607.
3. Hevia A, Delgado S, Margolles A, and Sanchez B. Application of density gradient for the isolation of the fecal microbial stool component and the potential use thereof. *Sci Rep.* 2015;5:16807.
4. Forti K, Ferroni L, Pellegrini M, Cruciani D, De Giuseppe A, Crotti S, et al. Molecular Characterization of Clostridium perfringens Strains Isolated in Italy. *Toxins (Basel).* 2020;12(10).
5. Livak KJ, and Schmittgen TD. Analysis of relative gene expression data using real-time quantitative PCR and the 2(-Delta Delta C(T)) Method. *Methods.* 2001;25(4):402-8.
6. Uzal FA, Glastonbury JR, Kelly WR, and Thomas R. Caprine enterotoxaemia associated with cerebral microangiopathy. *Vet Rec.* 1997;141(9):224-6.
7. Regan SB, Anwar Z, Miraflor P, Williams LB, Shetty S, Sepulveda J, et al. Identification of epsilon toxin-producing Clostridium perfringens strains in American retail food. *Anaerobe.* 2018;54:124-7.
8. Kolmogorov M, Yuan J, Lin Y, and Pevzner PA. Assembly of long, error-prone reads using repeat graphs. *Nat Biotechnol.* 2019;37(5):540-6.
9. Chen S, Zhou Y, Chen Y, and Gu J. fastp: an ultra-fast all-in-one FASTQ preprocessor. *Bioinformatics.* 2018;34(17):i884-i90.
10. Li H, and Durbin R. Fast and accurate long-read alignment with Burrows-Wheeler transform. *Bioinformatics.* 2010;26(5):589-95.

11. Li H. Aligning sequence reads, clone sequences and assembly contigs with BWA-MEM. *arXiv:13033997.* 2013.
12. Walker BJ, Abeel T, Shea T, Priest M, Abouelliel A, Sakthikumar S, et al. Pilon: an integrated tool for comprehensive microbial variant detection and genome assembly improvement. *PLoS One.* 2014;9(11):e112963.
13. Bankevich A, Nurk S, Antipov D, Gurevich AA, Dvorkin M, Kulikov AS, et al. SPAdes: a new genome assembly algorithm and its applications to single-cell sequencing. *J Comput Biol.* 2012;19(5):455-77.
14. Antipov D, Korobeynikov A, McLean JS, and Pevzner PA. hybridSPAdes: an algorithm for hybrid assembly of short and long reads. *Bioinformatics.* 2016;32(7):1009-15.
15. Hunt M, Silva ND, Otto TD, Parkhill J, Keane JA, and Harris SR. Circlator: automated circularization of genome assemblies using long sequencing reads. *Genome Biol.* 2015;16:294.
16. Darling AC, Mau B, Blattner FR, and Perna NT. Mauve: multiple alignment of conserved genomic sequence with rearrangements. *Genome Res.* 2004;14(7):1394-403.
17. Petkau A, Stuart-Edwards M, Stothard P, and Van Domselaar G. Interactive microbial genome visualization with GView. *Bioinformatics.* 2010;26(24):3125-6.
18. Linden JR, Telesford K, Shetty S, Winokour P, Haigh S, Cahir-McFarland E, et al. A Novel Panel of Rabbit Monoclonal Antibodies and Their Diverse Applications Including Inhibition of Clostridium perfringens Epsilon Toxin Oligomerization. *Antibodies (Basel).* 2018;7(4).
19. Linden JR, Flores C, Schmidt EF, Uzal FA, Michel AO, Valenzuela M, et al. Clostridium perfringens epsilon toxin induces blood brain barrier permeability via caveolae-dependent transcytosis and requires expression of MAL. *PLoS Pathog.* 2019;15(11):e1008014.
20. Rumah KR, Ma Y, Linden JR, Oo ML, Anrather J, Schaeren-Wiemers N, et al. The Myelin and Lymphocyte Protein MAL Is Required for Binding and Activity of Clostridium perfringens epsilon-Toxin. *PLoS Pathog.* 2015;11(5):e1004896.
21. Laaker C, Hsu M, Fabry Z, Miller SD, and Karpus WJ. Experimental Autoimmune Encephalomyelitis in the Mouse. *Curr Protoc.* 2021;1(12):e300.
22. Qin H, Yeh WI, De Sarno P, Holdbrooks AT, Liu Y, Muldowney MT, et al. Signal transducer and activator of transcription-3/suppressor of cytokine signaling-3 (STAT3/SOCS3) axis in myeloid cells regulates neuroinflammation. *Proc Natl Acad Sci U S A.* 2012;109(13):5004-9.
23. Recks MS, Stormanns ER, Bader J, Arnhold S, Addicks K, and Kuerten S. Early axonal damage and progressive myelin pathology define the kinetics of CNS histopathology in a mouse model of multiple sclerosis. *Clin Immunol.* 2013;149(1):32-45.
24. Pulido RS, Munji RN, Chan TC, Quirk CR, Weiner GA, Weger BD, et al. Neuronal Activity Regulates Blood-Brain Barrier Efflux Transport through Endothelial Circadian Genes. *Neuron.* 2020;108(5):937-52 e7.
25. Dobin A, Davis CA, Schlesinger F, Drenkow J, Zaleski C, Jha S, et al. STAR: ultrafast universal RNA-seq aligner. *Bioinformatics.* 2013;29(1):15-21.
26. Liao Y, Smyth GK, and Shi W. featureCounts: an efficient general purpose program for assigning sequence reads to genomic features. *Bioinformatics.* 2014;30(7):923-30.

27. Harrow J, Frankish A, Gonzalez JM, Tapanari E, Diekhans M, Kokocinski F, et al. GENCODE: the reference human genome annotation for The ENCODE Project. *Genome Res.* 2012;22(9):1760-74.
28. Ritchie ME, Phipson B, Wu D, Hu Y, Law CW, Shi W, et al. limma powers differential expression analyses for RNA-sequencing and microarray studies. *Nucleic Acids Res.* 2015;43(7):e47.
29. Robinson MD, McCarthy DJ, and Smyth GK. edgeR: a Bioconductor package for differential expression analysis of digital gene expression data. *Bioinformatics.* 2010;26(1):139-40.
30. Benjamini H HY. Controlling the False Discovery Rate: A Practical and Powerful Approach to Multiple Testing. *Journal of the Royal Statistical Society: Series B (Methodological).*57(1):289-300.
