# Supplementary material for: Epsilon toxin–producing Clostridium perfringens colonize the multiple sclerosis gut microbiome overcoming CNS immune privilege
Source: J Clin Invest. 2023 May 1;133(9):e163239. doi: 10.1172/JCI163239 (PMC10145940; doi:10.1172/JCI163239)
Supplement: Supplemental Table 1 [file jci-133-163239-s175.pdf]

Supplemental Table 1

Demographics of study participants, MS clinical phenotype, and MS disease modifying treatment.

	HEALTHY CONTROLS N=31	MULTIPLE SCLEROSIS N=31	TEST OF SIGNIFICANCE
AGE: MEAN (SD)	37.2 (13.7) yr	42.3 (12.5) yr	NS
SEX (% female)	64.5	80.6	NS
ANCESTRY/ETHNICITY (N)			
BLACK	3	4	
HISPANIC OR LATINO	4	6	NS
ASIAN	4	2	
WHITE	20	19	
RESIDENCE (N)			
URBAN	24	25	NS
SUBURBAN	6	5	
RURAL	1	1	
BMI: MEAN (SD)	25.12 (6.97)	26.85 (6.64)	NS
DIAGNOSIS			
RRMS	n/a	28	
SPMS	n/a	3	
PPMS	n/a	0	
MS TREATMENT*			
NAÏVE	n/a	9	
IFN-B	n/a	0	
COP-1	n/a	0	
DMF	n/a	1	
S1P1	n/a	2	
ANTI-VLA4	n/a	10	
ANTI-CD20	n/a	9	
ANTI-CD52	n/a	1	
NONE	n/a	1	

NS = Not significant; n/a = not applicable

- * Note that these numbers add to 33 due to 2 subjects that were transitioning from an S1P1 antagonist to an anti-CD20 and are thus counted twice.
- For age, a two-sample t-test yielded a p-value = 0.072

- For sex, the Fisher exact test statistic value was 0.2546. The result is *not* significant at $p < 0.05$.
- For ancestry/ethnicity the chi-square statistic was 1.2352. The p-value = 0.7446. The result is *not* significant at $p < 0.05$.
- For residence at time of fecal sample collection, the chi-square statistic was 0.1113. The p-value = 0.9459. The result is *not* significant at $p < .05$.
- For BMI, a two-sample t-test yielded a p-value of 0.318.
- Ancestry and ethnicity were self-reported. Residence is indicated at the time of fecal sample collection. Body-mass index (BMI) is the weight in kilograms divided by the square of the height in meters. Treatment naïve indicates MS subjects who were never on an MS disease modifying treatment. IFN = interferon beta, Cop = copolymer-1, DMF = dimethyl fumarate. S1P1 = an S1P1 agonist/antagonist, in this study all were Fingolimod. Anti-VLA4 = Natalizumab. Anti-CD20 = Rituximab or Ocrelizumab. Anti-CD52 = Alemtuzumab. None = patients who had previously received an MS disease modifying therapy but were not on active treatment for 6 months prior to fecal sample collection.
