# Supplementary material for: Epsilon toxin–producing Clostridium perfringens colonize the multiple sclerosis gut microbiome overcoming CNS immune privilege
Source: J Clin Invest. 2023 May 1;133(9):e163239. doi: 10.1172/JCI163239 (PMC10145940; doi:10.1172/JCI163239)
Supplement: Supplemental Table 2 [file jci-133-163239-s176.pdf]

Supplemental Table 2

Multivariable logistic regression using binary *etx* status as outcome confirms MS is independently associated with *etx* status, adjusting for sex and treatment.

	Characteristic	*OR*[1]	*95% CI*[2]	*p-value*
Group	HC	—	—	
	MS	25.2	4.46, 218	<0.001
	Female	—	—	
Gender	Male	0.68	0.14, 2.88	0.6
	No treatment	—	—	
Treatment	Treatment	0.29	0.04, 1.52	0.2

1 OR = Odds Ratio, 2 CI = Confidence Interval
